# Application of Mechanistic Modeling to Support Dose‐Relevant Decision Making for CAR‐T Cell Therapy in Oncology: A Systematic Scoping Review

**DOI:** 10.1111/cts.70756

**Published:** 2026-11-06

**Authors:** Dolapo Odujinrin, Graham McClelland

**Affiliations:** ^1^ Centre for Pharmaceutical Medicine Research, School of Cancer & Pharmaceutical Sciences, Faculty of Life Sciences & Medicine, King's College London London UK

**Keywords:** cancers, dose, mathematical modeling, mechanism‐based pharmacokinetics‐pharmacodynamics, model‐based drug development, multiscale modeling, quantitative pharmacology, translational medicine, translational pharmacokinetics‐pharmacodynamics

## Abstract

CAR‐T therapy has improved outcomes in selected relapsed or refractory hematologic malignancies. However, rational dose‐relevant decision application remains challenging because the administered (nominal) cell dose does not reliably predict in vivo exposure, toxicity, or clinical response. These relationships are not adequately characterized by empirical dose escalation alone. Mechanistic modeling may offer a biologically interpretable way to connect preclinical evidence and patient‐level CAR‐T posology, but its application remains incompletely defined. We conducted a systematic scoping review using JBI methodology and PRISMA‐ScR guidance, searching MEDLINE, Embase, Scopus, and Google Scholar (2010–2025). Eligible studies reported original CK/PD, PK/PD, QSP, PBPK/PD, stochastic, or agent‐based models for dose‐relevant application or regimen optimization. Nineteen studies met the inclusion criteria (13 mechanistic and 6 semi‐mechanistic models) spanning hematologic and solid‐tumor settings. Although these studies evaluated aspects of CAR‐T pharmacology, none provided prospectively validated clinical dose‐selection evidence. Overall, CAR‐T expansion appeared to be more influenced by tumor burden, antigen engagement, product‐specific attributes, lymphodepletion, and host or tumor‐site factors than by nominal dose. Solid‐tumor models highlighted delivery route, trafficking, biodistribution, and microenvironment barriers. Emerging in vivo CAR‐T platforms shifted dose considerations toward vector or mRNA dose and repeat dosing. Mechanistic modeling appears useful for framing CAR‐T dose‐relevant and regimen questions, but the current evidence is best interpreted as a taxonomy of model applications rather than proof of prospective clinical dose selection utility. Consistent with ICH M15 principles, future clinical, and regulatory application will require defined context of use, stronger validation, uncertainty assessment, and biological integration.

## Study Highlights

- What is the current knowledge on the topic?
  ○ CAR‐T dose‐relevant decision making remains product‐ and context‐specific and is often guided by empirical early‐phase clinical data. Although mechanistic and semi‐mechanistic models have been utilized to describe CAR‐T cellular kinetic exposure, tumor response, trafficking, lymphodepletion effects, and cytokine‐related toxicity, their prospective use to support the selection of dose levels for further clinical evaluation and inform regimen optimization remain limited.
- What question did this study address?
  ○ Which mechanistic and semi‐mechanistic CAR‐T models have been applied to dose‐ or regimen‐relevant questions in oncology, which biological processes and decision applications do they represent, and what evaluation approaches were reported in relation to their proposed use?
- What does this study add?
  ○ This systematic scoping review identified 19 studies spanning ODE‐based CK/PD, population CK/NLME, QSP, PBPK/PD, stochastic, agent‐based, translational PK/PD, and in vivo vector models. Among studies evaluating dose‐exposure relationships, the influence of nominal dose varied across products and disease settings. Tumor or target‐cell burden, antigen biology, product attributes, lymphodepletion, host immune status, and tumor‐site access were also modeled as potential modifiers of cellular exposure and response. No included study prospectively evaluated a clinical dose or regimen recommendation using an independent clinical dataset.
- How might this change clinical pharmacology or translational science?
  ○ The findings support a conceptual framework linking administered CAR‐T treatment, exposure determinants, cellular kinetics, pharmacodynamic outcomes, and potential model‐informed applications. Across the reviewed studies, modeling was used primarily to enhance mechanistic understanding, explore alternative scenarios, and inform trial design. However, evidence supporting regulatory applications, such as clinical dose or regimen selection, was limited. As models are increasingly expected to inform clinical or regulatory decision making, the question of interest, context of use, and intended influence of model outputs should be clearly defined a priori. Verification, validation, applicability assessments, and uncertainty analyses should be proportionate to the potential risk associated with model use. Moreover, when model outputs are intended to support high‐consequence decisions, independent clinical evaluation should be considered to ensure that the level of evidentiary support is commensurate with the potential impact of an incorrect decision.

## 1 Introduction

### 1.1 Clinical Context and Unmet Need

Chimeric antigen receptor T cell (CAR‐T) therapy has transformed cancer immunotherapy, with CD19‐ and BCMA‐targeted products achieving durable responses and occasional long‐term remissions in relapsed or refractory hematologic malignancies [1, 2, 3, 4, 5, 6]. During the period covered by this review, seven ex vivo autologous CAR‐T products had received United States Food and Drug Administration (FDA) approval for indications including B cell acute lymphoblastic leukemia (B‐ALL), diffuse large B cell lymphoma (DLBCL), mantle cell lymphoma (MCL), and multiple myeloma (Table 1) [14]. The clinical development landscape continues to evolve toward earlier treatment lines, additional hematologic indications, and solid tumors, although solid‐tumor applications remain constrained by antigen heterogeneity, trafficking barriers, immunosuppressive tumor microenvironments, and limited intratumoral persistence [15, 16, 17].

**TABLE 1:** Overview of FDA‐approved CAR‐T cell therapy products during the period covered in this scoping review and their dose‐regimen characteristics.

Product name	Manufacturer	Year	Target	Indication	Recommended dose	Dose‐regimen strategy	Key features
Kymriah (tisagenlecleucel) [7]	Novartis Pharmaceuticals Corporation	2017	CD19	Patients up to 25 years with r/r B‐cell precursor ALL; Adults with r/r LBCL after ≥ 2 lines including DLBCL, high‐grade B‐cell lymphoma, and DLBCL arising from FL; Adults with r/r FL after ≥ 2 lines	B‐ALL: ≤ 50 kg, 0.2–5.0 × 10^6^ CAR‐positive viable T cells/kg; > 50 kg, 0.1–2.5 × 10^8^ total CAR‐positive viable T cells Adult r/r LBCL and FL: 0.6–6.0 × 10^8^ CAR‐positive viable T cells	Hybrid: weight‐based for B‐ALL patients ≤ 50 kg; Fixed total‐cell range for > 50 kg B‐ALL and adult DLBCL/FL indications	First US FDA‐approved CAR‐T product; Autologous CD19 CAR‐T with 4‐1BB costimulation
Yescarta (axicabtagene ciloleucel) [8]	Kite Pharma Inc.	2017	CD19	Adults with high‐risk LBCL and r/r FL after ≥ 2 lines including DLBCL	Target dose: 2 × 10^6^ CAR‐positive viable T cells/kg body weight; maximum 2 × 10^8^ CAR‐positive viable T cells	Weight‐based with maximum total‐cell cap	Autologous CD19 CAR‐T with CD28 costimulation
Tecartus (brexucabtagene autoleucel) [9]	Kite Pharma Inc.	2020	CD19	Adults with r/r MCL; adults with r/r B‐cell precursor ALL	MCL: 2 × 10^6^ CAR‐positive viable T cells/kg; maximum 2 × 10^8^ CAR‐positive viable T cells ALL: 1 × 10^6^ CAR‐positive viable T cells/kg; maximum 1 × 10^8^ CAR‐positive viable T cells	Weight‐based with indication‐specific target dose and maximum total‐cell cap	Autologous CD19 CAR‐T. The ALL dose is lower than the MCL dose
Breyanzi (lisocabtagene maraleucel) [10]	Juno Therapeutics Inc., a Bristol‐Myers Squibb Company	2021	CD19	Adults with LBCL meeting labeled r/r criteria; adults with r/r CLL/SLL after ≥ 2 prior lines including BTK and BCL‐2 inhibitors (accelerated approval); adults with r/r FL after ≥ 2 lines; adults with r/r MCL after ≥ 2 lines including BTK inhibitor; adults with r/r MZL after ≥ 2 lines	LBCL after > = 2 lines: 50–110 × 10^6^ CAR‐positive viable T cells. LBCL after 1 line, CLL/SLL, FL, MCL, and MZL: 90–110 × 10^6^ CAR‐positive viable T cells. Defined 1:1 CD8:CD4 CAR‐positive viable T cell	Fixed total‐cell dose range with defined CD8/CD4 composition	Autologous CD19 CAR‐T with 4‐1BB costimulation and separately supplied CD8 and CD4 components
Abecma (idecabtagene vicleucel) [11]	Celgene Corporation, a Bristol‐Myers Squibb Company	2021	BCMA	Adults with r/r multiple myeloma after ≥ 2 prior lines of therapy including an immunomodulatory agent, a proteasome inhibitor, and an anti‐CD38 mAb	300–510 × 10^6^ CAR‐positive T cells	Fixed total‐cell dose range	Autologous BCMA‐directed CAR‐T. First US FDA‐approved cell‐based gene therapy for multiple myeloma
Carvykti (ciltacabtagene autoleucel) [12]	Janssen Biotech Inc.	2022	BCMA (dual epitope)	Adults with r/r multiple myeloma who have received ≥ 1 prior line of therapy including a proteasome inhibitor and an immunomodulatory agent	0.5–1.0 × 10^6^ CAR‐positive viable T cells/kg body weight; Maximum 1 × 10^8^ CAR‐positive viable T cells per single infusion	Weight‐based with maximum total‐cell cap	Autologous BCMA CAR‐T with two BCMA‐targeting single‐domain antibody binding domains
Aucatzyl (obecabtagene autoleucel) [13]	Autolus Limited	2024	CD19	Adults with r/r B‐cell precursor ALL.	410 × 10^6^ CD19 CAR‐positive viable T cells	Fixed total dose delivered as split dose; risk−/disease‐burden‐informed schedule based on bone marrow blast assessment	Autologous CD19 CAR‐T with fast off‐rate/low‐affinity CD19 binder design

*Note:* The year column reports initial FDA approval of the product, and several current labels have expanded indications beyond the original approval populations.

Abbreviations: ALL, acute lymphoblastic leukemia; B‐ALL, B‐cell acute lymphoblastic leukemia; BCMA, B‐cell maturation antigen; BCL‐2, B‐cell lymphoma 2; BTK, Bruton tyrosine kinase; CAR, chimeric antigen receptor; CAR‐T, chimeric antigen receptor T cell; CLL, chronic lymphocytic leukemia; DLBCL, diffuse large B‐cell lymphoma; FL, follicular lymphoma; LBCL, large B‐cell lymphoma; mAb, monoclonal antibody; MCL, mantle cell lymphoma; MZL, marginal zone lymphoma; PI, proteasome inhibitor; r/r, relapsed or refractory; SLL, small lymphocytic lymphoma.

*Source:* US Food and Drug Administration (2025) [7, 8, 9, 10, 11, 12, 13, 14] ‐ Respective product prescribing information (PI); and FDA.gov ‘Approved Cellular and Gene Therapy Products list’ with supporting documents (accessed 27 October 2025).

Despite these gains, CAR‐T therapy presents a complex clinical pharmacology paradigm as the administered (nominal) cell dose is only a partial determinant of therapeutic exposure that develops after infusion [18]. Unlike conventional small molecule drugs or biologics, CAR‐T cells can distribute, expand after antigen engagement, differentiate, contract, and persist in vivo to contribute substantially to overall treatment intensity [17, 19, 20]. Model‐based analyses of tisagenlecleucel, for example, described multiphasic cellular kinetics (CK), with rapid expansion, subsequent contraction, and prolonged persistence in some patients [21]. This may explain why simple nominal dose‐response reasoning is often insufficient. Expansion, efficacy, and inflammatory toxicity are likely determined by a complex interplay of factors beyond infused cell number, including tumor burden, antigen density, product composition, lymphodepletion, immune reconstitution, tissue trafficking, and toxicity‐management interventions [19, 20, 21, 22].

Many approved CAR‐T regimens still use fixed, weight‐based, or range‐based cell doses selected during early clinical development, while systematic dose–CK–response, and dose–toxicity characterization remains limited [19, 22]. Empirical dose escalation and nominal cell‐dose comparisons alone are therefore unlikely to identify regimens that balance efficacy, safety, manufacturing feasibility, and patient heterogeneity.

In this scoping review, *dose‐relevant decision making* refers to the application, or integration of clinical and experimental evidence with model‐derived insights to support the evaluation of CAR‐T dose levels and treatment regimens in oncology. This includes interpreting CK exposure–response relationships and assessing product‐, patient‐, and disease‐specific factors that may influence efficacy and toxicity. Regimen optimization refers to evaluating modifications to CAR‐T administration and associated treatment components that could influence CK, tumor‐site exposure, toxicity, or overall benefit‐risk. These components include lymphodepletion, dose scheduling, and fractionation, local versus systemic administration, repeat dosing or re‐infusion timing, bridging therapy, and toxicity interventions [22, 23].

### 1.2 Regulatory Context and Mechanistic Modeling

The final International Council for Harmonisation of Technical Requirements for Pharmaceuticals for Human Use (ICH) M15 guideline on model‐informed drug development (MIDD) provides a structured, question‐driven and risk‐informed framework for evaluating model‐derived evidence in the context of drug development and regulatory decision‐making [24]. Principally, the guidance highlights that model credibility should be judged against the question(s) being asked, not by model label alone. In addition to the question of interest, relevant considerations include model influence, consequence of a wrong decision, model risk, and impact, technical criteria, verification, validation, applicability, uncertainty assessment, and transparent reporting [24, 25, 26]. These considerations could be important for CAR‐T cell therapy, given that models differ to varying degrees in biological detail, data support, and intended use.

Mechanistic modeling employs mathematical representations of relevant biological processes and interactions to describe the dynamics of complex systems [27, 28]. By integrating available data, biological knowledge, and explicit assumptions regarding system components and their relationships, these models enable the characterization and simulation of system behavior. When adequately evaluated for a defined context of use, mechanistic models may also generate testable predictions [27, 28]. As such, they can support understanding of system dynamics, facilitate hypothesis generation, identify knowledge gaps, inform experimental and clinical study design, and contribute to decision making in drug discovery and development [27, 28, 29].

For the present review, models are classified pragmatically as mechanistic or semi‐mechanistic. Mechanistic models explicitly represent one or more CAR‐T‐relevant biological processes such as expansion, contraction, persistence, trafficking, tumor killing, cytokine signaling, or host immune interactions; as the dominant organizing structure. The equations governing these processes derive from biologically motivated hypotheses (mass‐action kinetics, receptor‐ligand binding, predator‐prey dynamics), and the parameters carry direct biological interpretability. Semi‐mechanistic models retain biologically‐derived structure but describe some components using population, statistical, or empirical relationships. Parameter estimation is performed within a population framework, and interindividual variability is characterized statistically rather than derived from mechanistic covariates alone. The 2024 FDA guidance for ex vivo‐manufactured CAR‐T cell products makes this framing clinically relevant [30], by directly emphasizing the assessment of pharmacokinetics (PK) or exposure, pharmacodynamics (PD) or response, and immunogenicity, while recognizing that CAR‐T cells can proliferate and persist in vivo. These recommendations support the rationale for mechanism‐based modeling in CAR‐T development that involve CK models, multiscale quantitative systems pharmacology (QSP) models, physiologically‐based pharmacokinetic (PBPK) or biodistribution models, cytokine‐toxicity models, tumor‐immune interaction models, stochastic or agent‐based models, and emerging models for in vivo CAR‐T delivery [17, 19, 20, 22, 23, 31, 32, 33, 34].

Several reviews have explored CAR‐T dose–response relationships [35], CK or computational modeling more broadly [20, 31, 36, 37, 38, 39]. To our knowledge, however, none has specifically examined how mechanistic and semi‐mechanistic models have been applied to support CAR‐T dose‐relevant decision making beyond empirical dose escalation alone. Here, our scoping review focuses on narrower clinical pharmacology questions: what models have been developed, what biology they represent, how they were evaluated, and how far their evidence can reasonably support clinical, or regulatory decision making. Hence, the objectives were to identify and classify eligible CAR‐T modeling studies, synthesize evidence on their mathematical frameworks and biological coverage, assess model evaluation approaches, and describe their alignment with contemporary MIDD credibility principles.

## 2 Methods

### 2.1 Protocol and Registration

The protocol for the scoping review (File S1) was not registered in a systematic review registry such as PROSPERO, as the research was designed to map mechanistic modeling approaches rather than evaluate direct clinical health outcomes. Nevertheless, the protocol was developed a priori, and key registry‐aligned practices were followed to support transparency and reproducibility, including the pre‐specified research questions, eligibility criteria, search strategy, screening procedures, and data‐extraction approach.

Notably, systematic methods were used, and the review was conducted in accordance with Joanna Briggs Institute methodology and the Preferred Reporting Items for Systematic Reviews and Meta‐Analyses extension for Scoping Reviews (PRISMA‐ScR) guidance [40, 41]. A PRISMA‐ScR checklist mapping all 22 items to specific manuscript sections was used (File S2).

### 2.2 Search Strategy

Literature searches were conducted in Ovid MEDLINE, Embase, and Scopus, with additionally targeted searching in Google Scholar, for records published from January 2010 to December 2025. Search strategies combined controlled vocabulary where available, and free‐text terms across four concept domains: cancer or malignancy; CAR‐T cell therapy; mechanistic or semi‐mechanistic modeling; and dose, exposure–response, or regimen optimization. Example terms included “neoplasms,” cancer*, tumor*, “chimeric antigen receptor,” “CAR T,” “CAR‐T,”, “cellular kinetics,” “CK,” “CK/PD,” “PK/PD,” “quantitative systems pharmacology,” “QSP,” “mathematical model,” “PBPK,” “dose selection,” “dose optimization,” “dosing,” and “exposure–response.” The Google Scholar searches used pre‐specified targeted queries. Given that the displayed hit counts were dynamic, yield was defined based on the number of records screened or exported, rather than the approximate totals shown in the interface. Full database‐specific search strings and the Google Scholar query details are provided in File S3.

### 2.3 Eligibility Criteria

Studies were eligible if they were full peer‐reviewed primary research articles published from 2010 to 2025, and reported a mechanistic or semi‐mechanistic model relevant to CAR‐T cell therapy in oncology. Eligible models included CK/PD, PK/PD, QSP, physiologically based PK/PD, stochastic, spatial, and agent‐based frameworks, provided they contained an interpretable biological or mechanistic structure. Cancer indication, CAR‐T target, product type, development phase, route of administration, and platform were unrestricted.

Eligible applications included CAR‐T dose selection, dose optimization, regimen optimization, dose‐exposure–response analysis, tumor‐response or toxicity‐response assessment, dosing schedule evaluation, lymphodepletion or preconditioning strategies, route of administration, local versus systemic delivery, and other regimen‐design scenarios. Exposure–response, tumor‐response, and toxicity‐response analyses were included only when linked to a dose‐relevant decision‐making or regimen‐optimization question.

Studies were excluded if they were reviews, perspectives, editorials, conference abstracts without full peer‐reviewed articles, regulatory assessments without original modeling, or studies in non‐cancer indications. Studies were also excluded if they: (1) evaluated non‐CAR‐T cell therapies; (2) investigated dual‐targeted, tandem, bicistronic, pooled, cocktail, or other multi‐antigen investigational CAR‐T products outside the pre‐defined product scope, unless model outputs or clinical data were reported separately for an eligible single‐target CAR‐T product; (3) used purely empirical, statistical, or descriptive models without a mechanistic biological framework; (4) provided insufficient methodological detail to allow model classification; (5) developed CAR‐T models without an explicit dose‐relevant decision making, dose‐response, dosing schedule, exposure‐response, or regimen‐optimization application.

### 2.4 Study Selection and Data Extraction

Two independent reviewers screened titles and abstracts against the eligibility criteria, with disagreements resolved by consensus. Full texts of potentially eligible articles were then assessed against the complete criteria, and data were extracted using a standardized form (File S4). Extracted items included first author, year, journal, cancer indication, target antigen, CAR‐T platform, model type, mathematical framework, biological components, dose or regimen application, model evaluation approach, and mechanistic or semi‐mechanistic classification.

### 2.5 Thematic Synthesis and Model Credibility Appraisal

A thematic synthesis approach was used to organize and compare evidence from the reviewed studies according to the mechanistic and quantitative clinical pharmacology domains relevant to CAR‐T dose‐relevant decision making and regimen optimization in oncology. The synthesis considered mathematical and computational modeling frameworks; exposure‐response relationships; product‐specific attributes; CK/PD relationships; biological process coverage; toxicity‐management approaches; model evaluation approaches; model credibility and reporting features. These areas were used to compare model structure, biological assumptions, decision relevance, quantitative outputs, translational applicability, and the extent to which each model was evaluated for its defined or implied use.

Model appraisal used a structured framework adapted from the Prediction model Risk Of Bias ASsessment Tool (PROBAST) [42]. Because PROBAST was developed for clinical prediction models rather than pharmacological mechanistic models, the assessment was treated as a credibility and reporting appraisal, not as a conventional prediction model risk‐of‐bias assessment. Four adapted domains were assessed: data and participants, biological inputs and assumptions, endpoints and outcomes, and analysis and model evaluation. The adaptations were informed by ICH M15 principles, QSP best practices, and published model evaluation guidance [43, 44].

## 3 Results

### 3.1 Study Selection and Characteristics

The search across three databases and Google Scholar identified 4704 records (Ovid MEDLINE: 57; Embase: 125; Scopus: 4097; Google Scholar: 425). After removing 244 duplicates, 4460 records were screened by title and abstract, of which 4368 were excluded. The remaining 92 articles underwent full‐text assessment, with 73 excluded for the following reasons: review/narrative articles (*n* = 24), non‐mechanistic models (*n* = 18), not dose‐ or regimen‐relevant (*n* = 16), and other cell therapies, not specific to CAR‐T (*n* = 15). This yielded 19 studies meeting all inclusion criteria that were included in the final synthesis. The PRISMA (2020) flow diagram is presented in Figure 1 [45].

**FIGURE 1:**
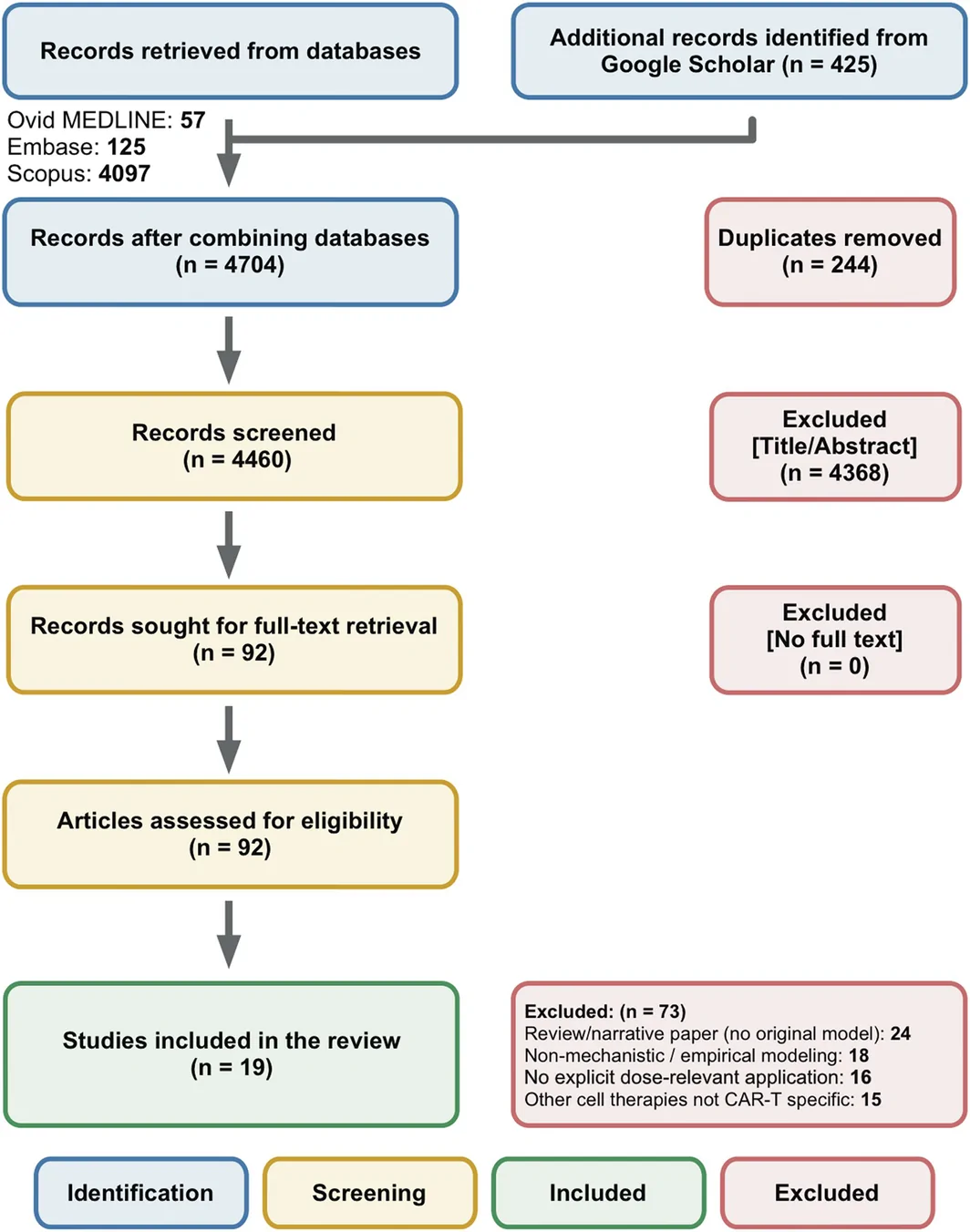
PRISMA flow diagram. PRISMA‐style flow diagram of study identification, screening, eligibility assessment, and inclusion for the systematic scoping review of mechanistic and semi‐mechanistic modeling studies to support dose‐relevant decision making for CAR‐T cell therapy in oncology. The flow diagram was adapted from the PRISMA 2020 reporting framework, and used with PRISMA‐ScR reporting guidance for scoping reviews. CAR‐T, chimeric antigen receptor T cell; Ovid MEDLINE, medical literature analysis and retrieval system online; PRISMA, preferred reporting items for systematic reviews and meta‐analyses; PRISMA‐ScR, preferred reporting items for systematic reviews and meta‐analyses for scoping reviews.

The 19 reviewed studies were published between 2019 and 2025, reflecting the emerging nature of this field. The chronological distribution by model class and the major model‐application clusters are summarized in Figure 2A. Thirteen studies (68%) were classified as mechanistic (M) and 6 (32%) as semi‐mechanistic (SM) models. Table 2 summarizes the 19 reviewed studies; the reviewer‐assigned model class, modeling framework, parameterization, and reported evaluation, dose‐relevant applications, and regimen optimization, or treatment‐strategy application, as reported by the publication authors. The reviewed studies spanned hematologic malignancy, solid tumor, and solid tumor‐relevant modeling contexts, but the empirical support differed across these settings. Many solid tumor and simulation‐based design space models were supported primarily by preclinical, translational, literature‐derived, or theoretical data, whereas comparatively fewer studies incorporated retrospective clinical CAR‐T datasets for model calibration or evaluation.

**FIGURE 2:**
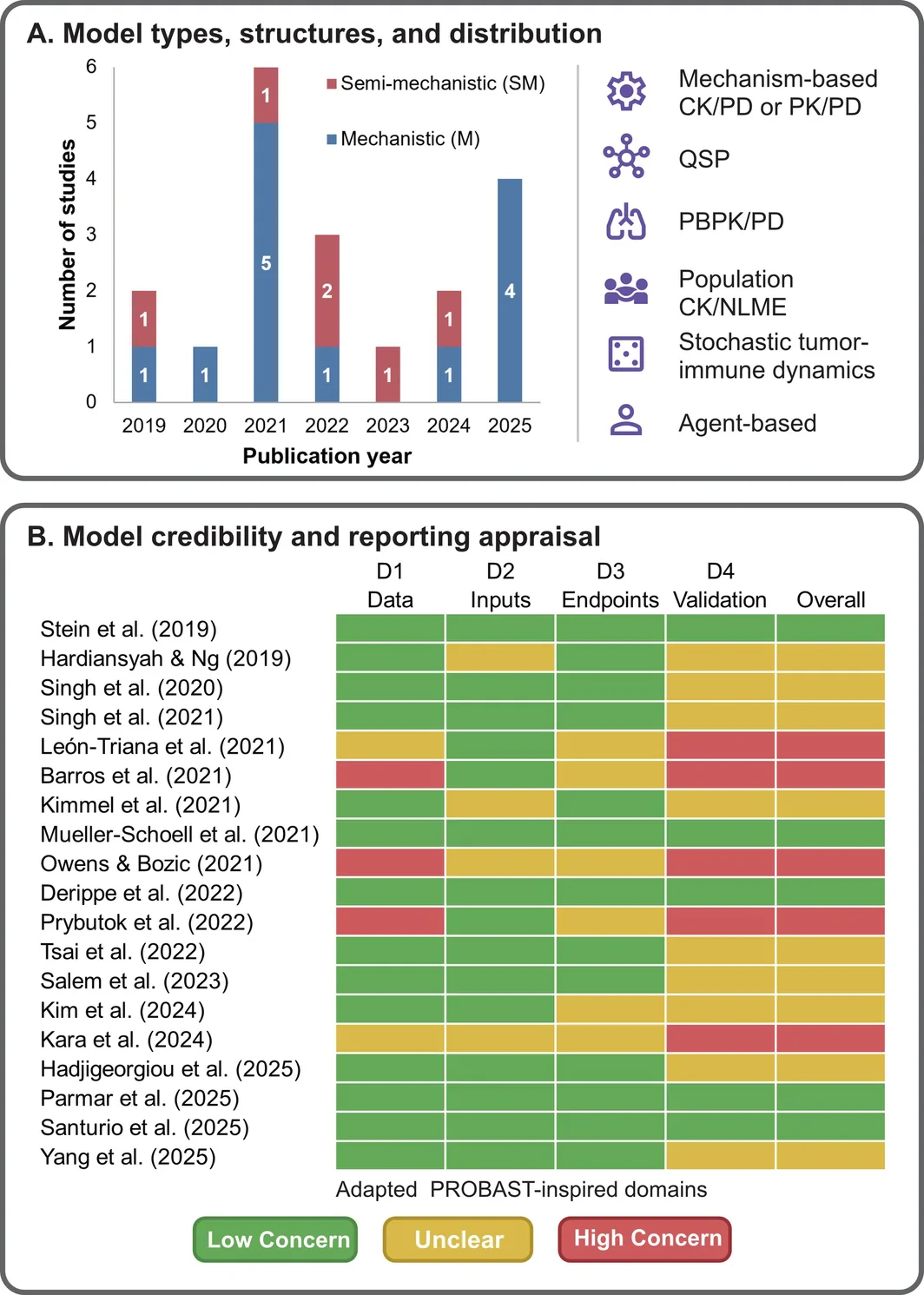
Model types, publication‐year distribution, and credibility and reporting appraisal of mechanistic and semi‐mechanistic CAR‐T models in oncology. (A) Model types, structures, and publication‐year distribution. Annual distribution of the 19 studies included in the systematic scoping review (2019–2025), classified as mechanistic (M; *n* = 13) or semi‐mechanistic (SM; *n* = 6) according to the review‐specific classification criteria. The accompanying list identifies mathematical modeling approaches represented across the studies, including mechanism‐based CK/PD or PK/PD models, QSP, PBPK/PD models, population CK/NLME models, stochastic tumor‐immune dynamics, and agent‐based models. These approaches are not necessarily mutually exclusive, and their listing does not indicate relative frequency. (B) Model credibility and reporting appraisal. Study‐level assessment using four adapted PROBAST‐inspired domains: D1, Data; D2, Inputs; D3, Endpoints; and D4, Validation. Individual domain ratings and overall assessments are presented as low concern (green), unclear (yellow), or high concern (red). Ratings reflect reviewer judgments and do not imply regulatory qualification or prospective clinical utility. CAR‐T, chimeric antigen receptor T‐cell therapy; CK, cellular kinetics; M, mechanistic; NLME, nonlinear mixed effects; PBPK, physiologically based pharmacokinetic; PD, pharmacodynamics; PK, pharmacokinetics; PROBAST, Prediction model Risk Of Bias ASsessment Tool; QSP, quantitative systems pharmacology; SM, semi‐mechanistic.

**TABLE 2:** Applications of mechanistic and semi‐mechanistic models to dose‐relevant decision‐making for CAR‐T therapy in oncology.

No.	Study; model class and framework	Disease/experimental setting	Parameterization and reported evaluation	Dose‐relevant applications	Regimen‐ or treatment‐strategy application
1	Stein et al. (2019) [21]; SM; population NLME CK model of expansion, contraction, and persistence	B‐ALL; CD19; tisagenlecleucel	Pooled clinical CK data; population and individual fits and VPCs. Internal evaluation only; no independent external dataset	Direct: evaluated dose–exposure and covariate relationships; no association between infused dose and *C*_max_ or other model parameters was detected	Evaluated whether tocilizumab or corticosteroids affected expansion. This was a supportive‐care question; alternative CAR‐T or rescue‐treatment doses and schedules were not optimized
2	Hardiansyah & Ng (2019) [32]; M; QSP model of CAR‐T cells, malignant/normal B cells and inflammatory cytokines	Advanced CLL; CD19	Literature‐informed model fitted principally to longitudinal data from two patients; selected CK and cytokine summary metrics were compared with two external published datasets. This was aggregate‐data comparison, not independent patient‐level validation	Direct but exploratory: simulated effects of infused dose and baseline disease burden on CAR‐T and cytokine profiles; conclusions were limited by sparse calibration data and the restricted simulated range	No alternative schedule, route, redosing, or conditioning regimen was compared; implications for personalized dosing and CRS risk were hypothesis‐generating
3	Singh et al. (2020) [33]; M; multiscale cell‐level PD and mouse PBPK/PD framework	Preclinical CAR‐T constructs targeting BCMA, CD19, EGFR, or HER2	Multiple in vitro and xenograft datasets; physiological and system parameters partly fixed from literature; multi‐dataset fitting, in vitro–in vivo potency comparison and global sensitivity analysis. No clinical evaluation	Direct, preclinical: varied single intravenous dose and tumor burden and predicted a steep dose–exposure relationship; also evaluated CAR affinity/density and antigen abundance as exposure–response determinants	Compared single and multiple dosing regimens and intravenous versus intratumoral delivery; the model captured tumor‐growth profiles under both schedules and routes. Construct−/target‐design optimization (affinity, density, antigen abundance) was also a principal application
4	Singh et al. (2021) [34]; M; multiscale translational anti‐BCMA CK/PD model	Relapsed/refractory MM; BCMA; bb2121/idecabtagene vicleucel	Sequential integration of in vitro, xenograft and phase I clinical CK/biomarker data; sensitivity analysis and virtual‐patient simulations. Results were compared with later reported clinical observations, but not prospectively evaluated in an independent patient‐level dataset	Direct: single‐infusion simulations predicted a steep dose–exposure–response relationship and an apparent threshold near 150 × 10^6^ cells, above which additional exposure and response gains were limited. The threshold is product‐ and model‐specific	Multiple infusions were proposed as a future application but were not simulated; the study compared single‐infusion dose levels rather than schedules
5	León‐Triana et al. (2021) [46]; M; mechanistic ODE model of CAR‐T, leukemic and healthy B cell compartments and a side‐effect proxy	B‐ALL; CD19	Literature‐informed theoretical model; analytical derivations and sensitivity/scenario analyses with qualitative comparison to reported clinical behavior. No patient‐level calibration or independent evaluation.	Direct, theoretical: predicted that initial CAR‐T dose had limited influence on peak expansion and outcome in antigen‐rich conditions, whereas target‐cell availability and CAR‐T persistence were more influential.	Explored repeat CAR‐T administration as a theoretical strategy for controlling relapse and examined the role of CAR‐T lifetime/persistence.
6	Barros et al. (2021) [47]; M; three‐population ODE model (tumor, effector CAR‐T and memory CAR‐T; CARTmath)	Immunodeficient‐mouse hematologic‐tumor models; CD123/HDLM‐2 and CD19/Raji	Selected parameters calibrated to two published in vitro/in vivo datasets; remaining uncertain parameters explored through simulation and a virtual population. No clinical evaluation	Direct, preclinical: mapped tumor‐burden‐dependent effective‐dose regions and predicted loss of response below model‐specific CAR‐T dose thresholds	Compared single and fractionated dosing and explored tumor rechallenge and CAR‐T/indoleamine 2,3‐dioxygenase‐inhibitor (1‐methyl‐tryptophan) combination scenarios
7	Kimmel et al. (2021) [48]; M; deterministic–stochastic ecological model of normal T cell, CAR‐T and tumor competition	LBCL; CD19; clinical population‐level data	Parameterized with published longitudinal CAR‐T and tumor data; model predictions were compared with reported progression‐free survival. No independent external patient‐level evaluation	Indirect: explained tumor extinction through CAR‐T expansion relative to tumor burden, normal‐T cell competition, killing rate and carrying capacity; it did not establish a clinical CAR‐T dose threshold	Simulated a second CAR‐T infusion with or without renewed lymphodepletion and predicted that repeat lymphodepletion was needed to restore expansion
8	Mueller‐Schoell et al. (2021) [49]; SM; population QSP model of CAR‐T phenotypes and metabolic tumor burden	Relapsed/refractory NHL; CD19	Clinical data from 19 patients plus literature/fixed values for weakly identifiable parameters; internal model assessment. The composite score cut‐off was derived in the same cohort and requires external validation	Indirect: the observed naïve‐phenotype (T_N_) *C*_max_, normalized to baseline metabolic tumor burden, was translated into a clinical composite score (CCS_TN_) that correlated with the model‐estimated expansion parameter; it supported exposure‐ stratification, not dose selection	No regimen was compared; proposed application was early risk prediction and monitoring
9	Owens and Bozic (2021) [50]; M; ODE tumor–immune–chemotherapy–CAR‐T model	Generic cancer scenarios informed by hematologic and solid‐tumor literature	Literature‐informed theoretical parameterization; numerical treatment simulations and sensitivity analysis. No clinical calibration or independent validation	Direct, theoretical: estimated the minimum CAR‐T dose needed for remission as a function of killing efficiency and chemotherapy preconditioning	Compared chemotherapy timing, total exposure and delivery schedules, showing that schedules with the same chemotherapy amount and CAR‐T dose could yield different outcomes
10	Derippe et al. (2022) [51]; SM; population mechanism‐based PK/PD model of lymphodepletion, host lymphocytes, IL‐7 and UCART19	Adult relapsed/refractory B‐ALL; CD19; allogeneic UCART19	CALM phase I clinical data with literature‐informed/fixed parameters; numerical predictive checks reproduced expansion frequencies overall and by alemtuzumab‐dose group. No independent external validation	Indirect for UCART19 dose: because cell dose was not associated with expansion, a common nominal dose was used in the mechanistic model; the analysis instead quantified lymphodepletion, IL‐7 and host‐versus‐graft effects on exposure	Direct conditioning application: compared fludarabine/cyclophosphamide with versus without alemtuzumab and supported optimization of future lymphodepletion regimens
11	Prybutok et al. (2022) [52]; M; spatial agent‐based CARCADE model	Generic solid‐tumor dish and vascularized‐tissue contexts	Biological rules and parameters informed by published studies; model was not trained to one experiment. Qualitative reproduction of experimental trends and extensive multidimensional parameter sweeps; no clinical evaluation.	Direct, in silico: varied CAR‐T dose with CD4:CD8 ratio, CAR affinity and tumor/healthy‐cell antigen density; dose increased CAR‐T numbers but, in tissue simulations, killing/selectivity depended more strongly on affinity and antigen context.	No schedule was evaluated. The primary application was multidimensional product/target design, not regimen optimization.
12	Tsai et al. (2022) [53]; SM; mouse minimal PBPK/PD models of pleural and liver tumors	Pleural mesothelin‐positive and liver CEA‐positive mouse tumor models	Physiological/literature values and naïve‐pooled fitting to digitized mouse CK and tumor data; sensitivity and route simulations. No clinical evaluation.	Direct, preclinical: predicted a tumor‐compartment CAR‐T threshold for proliferation/efficacy and quantified how route altered early tumor exposure.	Compared intrapleural with intravenous delivery and hepatic‐artery with portal‐vein/intravenous delivery; local delivery near the tumor produced the greatest predicted benefit.
13	Salem et al. (2023) [54]; SM; multiscale CK/PD model of CD4/CD8 subsets and memory/effector immunophenotypes	DLBCL and adult/pediatric ALL; CD19; liso‐cel and CAT19/FMC63‐based products	In vitro data plus published, partly digitized clinical CK and immunophenotype data; sequential model fits. Evaluation was limited by summary‐level covariates and small immunophenotype datasets; no independent external validation.	Indirect: quantified how CD4:CD8 composition, phenotype, CAR affinity and potency translate a nominal dose into differential expansion and persistence; it did not prospectively select a dose.	No dose schedule was compared. The intended future application was optimization of product composition, which should be distinguished from regimen optimization.
14	Kim et al. (2024) [55]; SM; translational cell‐level PK/PD model of mRNA‐loaded nanoparticles and transient CAR expression	Preclinical in vivo generation of CD19 CAR‐T cells	Published in vitro and mouse data plus nanoparticle PK/binding information; parameter precision, individual fits, VPCs and global sensitivity analysis. No human evaluation.	Direct, preclinical: quantified the relationship between nanoparticle‐encapsulated mRNA dose and transient surface CAR expression.	Simulated dose amounts and intervals from every 3 to 14 days; a 2‐fold dose every 5 days was proposed to sustain CAR expression in the model.
15	Kara et al. (2024) [56]; M; ODE model of antigen‐positive/negative tumor cells, CAR‐T cells and bystander cells	Preclinical antigen‐heterogeneous solid tumors	Sequential calibration to published mouse data, literature‐informed parameters, Sobol sensitivity analysis and virtual‐patient simulations. Cross‐species parameter borrowing limited predictive scope; no clinical validation.	Direct, preclinical: simulated CAR‐T dose across antigen‐positive fractions and predicted that dose escalation alone was insufficient when substantial antigen‐negative disease remained.	Explored cyclophosphamide‐associated bystander activity and increased bystander‐cell killing as adjunct strategies; no alternative CAR‐T schedule or specific drug regimen was optimized.
16	Hadjigeorgiou et al. (2025) [57]; M; PBPK model of CAR‐T/endogenous immune‐cell trafficking, tumor response and vascular/stromal normalization	Mouse GBM; EGFRvIII CAR‐T/anti‐VEGF; additional desmoplastic‐tumor scenarios	Calibrated/compared with tumor‐growth and immune‐infiltration data from two GBM mouse models and selected literature data. No clinical evaluation; preclinical comparisons should not be described as prospective external clinical validation.	Direct, preclinical: simulations predicted that vascular normalization could reduce the infused CAR‐T amount required for tumor control by approximately fivefold.	Compared anti‐VEGF‐CAR‐T sequencing, timing of a second infusion, local versus systemic delivery, anti‐VEGF‐secreting CAR‐T cells and combined vascular/stromal normalization.
17	Parmar et al. (2025) [58]; M; clinical PBPK/PD model of effector/memory CAR‐T cells, host T cells, antigen heterogeneity and tumor response	Phase I solid‐tumor studies; mesothelin, GPC3 and PSMA/TGF‐β‐targeted products	Digitized individual CK, antigen‐expression and categorical response data from four phase I programs (45 patients); simultaneous characterization of the included datasets. No clearly independent held‐out clinical evaluation was reported.	Direct/interpretive: characterized CK and response across clinical dose ranges and identified baseline tumor burden and antigen‐positive fraction as important expansion determinants; it did not establish a universal dose.	Compared cohorts with and without lymphodepletion and quantified its predicted effects on host competition, expansion and persistence.
18	Santurio et al. (2025) [59]; M; multilayer ODE model of CAR‐T/tumor dynamics, antigen‐negative relapse, macrophage activation and IL‐6	Published patient data across CD19‐positive hematologic malignancies	Layer‐wise fitting to 25 published patient time courses, including IL‐6 data for 15; scenario and sensitivity analyses. No independent external or prospective clinical validation.	Direct, simulation‐based: 10‐ and 100‐fold changes in dose and baseline tumor burden were simulated; smaller doses delayed/reduced the predicted IL‐6 peak with little change in modeled remission, but this requires clinical confirmation.	Generated hypotheses for tumor debulking and inhibition of macrophage activation, particularly CD40–CD40L signaling; it did not optimize a specific drug dose or intervention schedule.
19	Yang et al. (2025) [60]; M; multiscale QSP platform linking CAR–antigen interactions, biodistribution, phenotype transitions, tumor heterogeneity and virtual patients	Solid tumors; CLDN18.2/LB1908, with cross‐target applications to HER2, EGFR, GPC3 and mesothelin	Stepwise calibration and evaluation with multimodal in vitro and mouse data, including held‐out experimental data, plus comparison with published data for other targets and sensitivity/KDE analyses. LB1908 clinical dose projections were not independently prospectively validated.	Direct: virtual patients were simulated at 50, 150 and 250 million cells and under weight‐based versus flat dosing; predicted dose–response varied substantially between patients.	Compared single‐dose, equal fractionation and step‐up schedules; predicted efficacy was similar at the same total dose, without prospective clinical confirmation.

*Note:* Model classes (M and SM) are reviewer‐assigned pragmatic structural descriptors and do not represent formal ICH M15 model categories. Direct dose relevance indicates that an administered cell or vector dose, or a defined dose range, was explicitly evaluated or varied. Indirect dose relevance indicates that the model examined determinants that could inform interpretation of exposure–response relationships without explicitly selecting, comparing, or varying an administered dose. Regimen optimization was assigned only when a modifiable treatment feature such as schedule, fractionation, dosing interval, redosing, route of administration, conditioning, or concomitant therapy was directly compared. Product or construct optimization and risk characterization were retained as distinct treatment‐strategy applications and were not classified as regimen optimization. Model‐evaluation terminology describes the activities reported and does not imply formal validation or regulatory qualification. Comparisons with published aggregate data were distinguished from evaluations using independent patient‐level datasets. No included study prospectively validated a clinical dose or regimen recommendation using an independent clinical dataset.

Abbreviations: AUC, area under the concentration‐time curve; B‐ALL, B cell acute lymphoblastic leukemia; BCMA, B cell maturation antigen; bb2121, anti‐BCMA CAR‐T product idecabtagene vicleucel; CAR‐T, chimeric antigen receptor T cell; CEA, carcinoembryonic antigen; CK, cellular kinetics; CLL, chronic lymphocytic leukemia; *C*_max_, maximum concentration; CRS, cytokine release syndrome; DLBCL, diffuse large B cell lymphoma; EGFR, epidermal growth factor receptor; FAP, fibroblast activation protein; HER2, human epidermal growth factor receptor 2; HSCs, hematopoietic stem cells; ICH, International Council for Harmonization of Technical Requirements for Pharmaceuticals for Human Use; IL‐7, interleukin‐7; KDE, kernel density estimation; LBCL, large B cell lymphoma; LDC, prior lymphodepletion chemotherapy; M, mechanistic; MM, multiple myeloma; mPBPK, minimal physiologically based pharmacokinetic; mRNA, messenger ribonucleic acid; NHL, non‐Hodgkin lymphoma; NLME, nonlinear mixed‐effects; NOS, not otherwise specified; NP, nanoparticle; ODE, ordinary differential equation; OS, overall survival; PBPK, physiologically based pharmacokinetic; PD, pharmacodynamic(s); PFS, progression‐free survival; PK, pharmacokinetic(s); PSMA, prostate‐specific membrane antigen; QSP, quantitative systems pharmacology; r/r, relapsed/refractory; SM, semi‐mechanistic; VPCs, visual predictive checks.

### 3.2 Mathematical Frameworks

The 19 reviewed studies represented a diverse set of mathematical frameworks, reflecting the multifaceted pharmacology of CAR‐T, and the range of dose‐relevant applications and regimen‐optimization questions examined (Figure 2A). Ordinary differential equation (ODE)‐based systems were the most common mathematical backbone, either as standalone CK/PD or PK/PD models or as components of multiscale, QSP, PBPK/PD, stochastic, or population modeling frameworks [32, 33, 34, 35, 36, 37, 38, 39, 40, 41, 42, 43, 44, 46, 47, 48, 50, 56, 59, 60]. PBPK or minimal PBPK/PD models were utilized mainly for biodistribution, tissue‐exposure, and delivery‐route questions, particularly in solid‐tumor or regional‐delivery contexts [53, 57, 58]. QSP models were utilized when the modeling question required integration across biological scales, including CAR‐T CK, tumor dynamics, cytokine/toxicity mechanisms, biodistribution, or virtual‐patient response [32, 49, 60].

Multiscale systems PK/PD models addressed related questions of product design and translation by linking CAR affinity and antigen abundance to tumor killing, CAR‐T expansion, cytokine release, and dose‐exposure‐response relationships [33, 34]. Prybutok et al. [52] extended the spatial agent‐based representation of cells and dynamic environments (ARCADE) modeling framework to include CAR‐T cell agents, creating CAR‐T cell ARCADE (CARCADE), which provided a complementary in silico perspective by exploring how CAR‐T cell dose, CD4+/CD8+ ratio, CAR‐antigen affinity, cancer‐cell antigen expression, and healthy‐cell antigen expression influenced simulated efficacy, selectivity, cytokine production, and spatial treatment performance [52].

The reviewed studies exhibited marked variation in model complexity. Approaches included ODE‐based models of tumor CAR‐T cell interactions, solid tumor models incorporating antigen heterogeneity and bystander effects, minimal and tissue distribution PBPK/PD models, clinical PBPK‐PD models accounting for lymphodepletion and antigen‐positive tumor burden, and multiscale QSP models with virtual patient simulations. Across these modeling frameworks, a wide range of biological and clinical processes were represented. These included CAR‐T cell expansion, contraction, persistence, and tissue distribution; tumor‐cell killing; antigen‐positive and antigen‐negative tumor dynamics; antigen heterogeneity and bystander killing; host immune responses and lymphodepletion effects; vascular and stromal normalization; disease relapse; macrophage‐mediated cytokine release; and patient‐level treatment response [46, 53, 56, 57, 58, 59, 60]. Within the reviewer‐assigned semi‐mechanistic category, models generally combined biologically motivated structure with empirical, population, translational, or platform‐specific components. Depending on the study context, these approaches supported clinical CK characterization, cross‐scale interpretation, or exploration of product‐ and platform‐specific determinants of CAR‐T exposure and response [21, 49, 51, 53, 54, 55]. Taken together, these frameworks were complementary rather than hierarchical, with their relevance depending on the biological process, data source, and modeling question addressed.

### 3.3 Dose‐Relevant Decision Making

Across the reviewed studies, the relationship between nominal infused dose and subsequent CAR‐T cellular exposure varied across models, products, and disease settings. Studies reported or predicted absent, limited, nonlinear, or threshold‐like relationships. In a population analysis of pooled data from two phase II B‐ALL studies, Stein et al. [21] found no detectable relationship between tisagenlecleucel dose and *C*_max_ or any estimated model parameter. In a theoretical B‐ALL model, León‐Triana et al. [46] predicted that a 10‐fold change in initial CAR‐T cell dose altered maximum expansion by approximately 6% and time to maximum expansion by approximately 3 days under the simulated antigen‐rich conditions. The model was not calibrated to patient‐level data [46]. In a multiscale anti‐BCMA analysis integrating in vitro, xenograft, and phase I clinical data, Singh et al. [34] predicted that *C*_max_ was more sensitive to baseline tumor burden than to administered dose. The modeled dose‐exposure‐response relationship approached an apparent plateau near 150 × 10^6^ cells, above which additional exposure and response gains were limited. This threshold was model‐ and product‐specific and was not established as a clinical dose threshold [34]. Hardiansyah and Ng [32] modeled dose and baseline tumor burden as joint determinants of CAR‐T expansion, B cell depletion, and cytokine profiles. The model was fitted principally to longitudinal data from two patients with CLL, and comparisons with other studies used published aggregate metrics rather than an independent patient‐level dataset [32].

Preclinical and theoretical models evaluated dose jointly with product and disease characteristics. Barros et al. [47] mapped tumor‐burden‐dependent effective‐dose regions and predicted loss of response below model‐specific dose thresholds in immunodeficient mouse settings [47]. Singh et al. [33] evaluated dose and tumor burden together with CAR affinity and density, antigen abundance, and effector‐to‐target conditions. In solid‐tumor design‐space analyses, Prybutok et al. [52] varied dose with CD4 composition, CAR affinity, and tumor‐ and healthy‐cell antigen density, whereas Kara et al. [56] predicted that increasing dose alone did not overcome substantial antigen‐negative disease [52, 56]. These findings were based on preclinical or in silico analyses and did not establish clinical dose thresholds.

Several studies had indirect dose relevance because they evaluated determinants of exposure without comparing administered CAR‐T doses. In a 19‐patient non‐Hodgkin lymphoma cohort, Mueller‐Schoell et al. [49] related model‐estimated expansion capacity to prior autologous stem‐cell transplantation and the Day 7 CAR‐positive CD4:CD8 ratio. The study also derived a clinical composite score based on maximum naïve‐phenotype CAR‐T concentration normalized to baseline metabolic tumor burden. The score was developed and assessed in the same cohort and was not independently evaluated [49]. In the allogeneic UCART19 analysis, administered cell dose was not associated with expansion and was standardized in the mechanistic model. Derippe et al. [51] instead modeled the effects of lymphodepletion‐associated IL‐7 availability, host T cell abundance, and host‐mediated elimination on UCART19 expansion and persistence. Salem et al. [54] modeled CAR‐T subset composition and immunophenotypic transitions as potential determinants of expansion and persistence but did not directly compare administered doses.

In solid‐tumor models, effective tumor‐site exposure was evaluated in relation to administration route, trafficking, and the tumor microenvironment. Mouse minimal PBPK/PD analyses by Tsai et al. [53] predicted that intrapleural or hepatic‐artery administration increased initial tumor exposure and accelerated proliferation relative to the systemic comparators evaluated. In the pleural model, local administration reached a modeled tumor‐compartment exposure threshold at a low dose for which intravenous administration did not produce expansion or tumor regression. These results were specific to the modeled mouse systems and routes [53]. Hadjigeorgiou et al. [57] predicted that anti‐VEGF‐mediated vascular normalization could increase tumor infiltration and reduce the CAR‐T dose required for tumor control by approximately 5‐fold in mouse glioblastoma multiforme (GBM) models. Neither study established a clinical dose reduction or route recommendation.

Using digitized data from four phase I solid‐tumor programs, Parmar et al. [58] characterized relationships among administered dose, baseline tumor burden, antigen‐positive tumor fraction, lymphodepletion, tissue distribution, and CK. Model simulations predicted that initial tumor burden and antigen‐positive fraction influenced expansion; no clearly independent, held‐out clinical evaluation was reported [58]. Yang et al. [60] used virtual‐patient simulations to compare dose levels, flat versus weight‐based dosing, and single, equal‐fraction, and step‐up schedules. Although the modeling platform was evaluated using held‐out experimental data, its clinical dose and regimen projections were not prospectively evaluated in an independent clinical dataset [60].

Across these heterogeneous analyses, nominal dose was represented as the initial treatment input rather than the sole determinant of subsequent cellular exposure. The models additionally represented target‐cell burden, antigen biology, product attributes, lymphodepletion, host immunity, administration route, and tissue trafficking as potential exposure–response determinants. Their relative contributions varied by model, product, and disease setting, and no universal dose–exposure relationship or clinically validated dose threshold was established.

### 3.4 Integrated Exposure–Response Evidence Framework

The reviewed evidence was synthesized into a five‐component framework (Figure 3A) linking administered treatment inputs to model‐represented determinants, in vivo cellular exposure and distribution, PD and clinical outcomes, and potential model‐informed applications. The framework is a cross‐study evidence map and not a single integrated model, validated causal pathway, or clinical decision algorithm.

- Component 1, *Treatment inputs*: Administered inputs included viable CAR‐positive cell dose or vector/mRNA amount, flat or weight‐based dosing, route of administration, schedule, fractionation, and redosing.
- Component 2, *Exposure and response determinants*: Model‐represented determinants included product or construct attributes, such as viability, CAR‐positive fraction, CAR density and affinity, phenotype, potency, and cellular composition. Patient and host factors included immune status and reconstitution; disease factors included tumor or target‐cell burden, antigen density and heterogeneity, and the tumor microenvironment; and treatment‐context factors included lymphodepletion and bridging or concomitant therapy.
- Component 3, *In vivo cellular exposure and distribution*: CK was characterized by expansion, *C*_max_, *T*_max_, AUC, expansion rate or doubling time, contraction, persistence, and terminal half‐life. Some models also represented blood, tissue, or tumor‐site distribution.
- Component 4, *Pharmacodynamic and clinical outcomes*: Modeled pharmacodynamic readouts included tumor or target‐cell dynamics, B‐cell aplasia, cytokines, and other biomarkers. Clinical outcomes included response, response durability, and progression‐free survival where evaluated. Safety modeling focused mainly on CRS‐related outcomes or proxies; explicit immune effector cell‐associated neurotoxicity syndrome (ICANS) mechanisms and clinical predictions were not identified, representing an evidence gap.
- Component 5, *Potential model‐informed applications*: Applications included exploring dose levels or ranges; comparing schedules, fractionation, redosing, routes, and lymphodepletion strategies; and generating hypotheses about product or construct design, risk mitigation, and trial design. These applications were predominantly exploratory and hypothesis‐generating; none constituted prospectively validated clinical dose selection or established benefit–risk thresholds.

**FIGURE 3:**
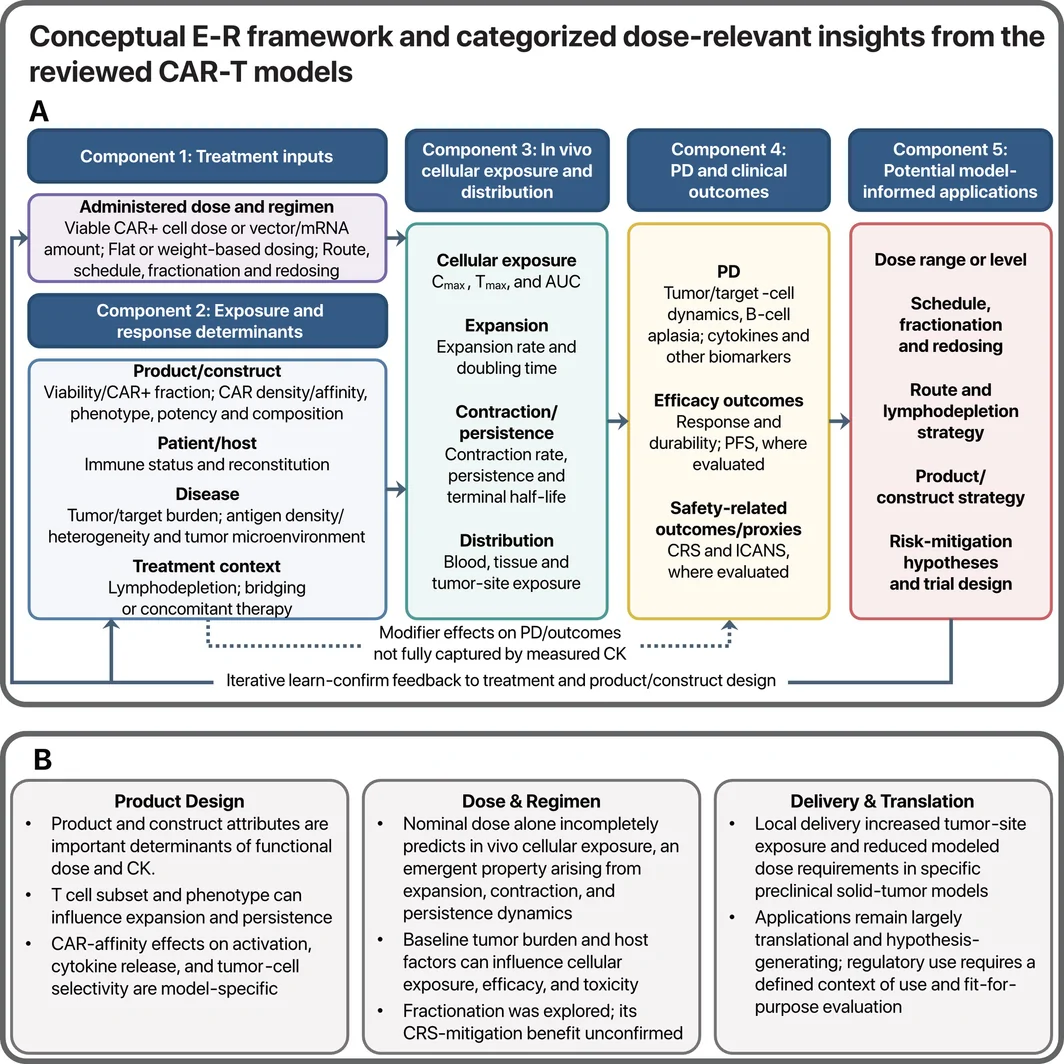
Conceptual exposure–response framework and categorized dose‐relevant insights from mechanistic and semi‐mechanistic CAR‐T models in oncology. (A) Conceptual exposure–response framework. Cross‐study synthesis linking five components relevant to CAR‐T dose‐relevant decision making: (1) administered treatment inputs; (2) exposure and response determinants; (3) in vivo exposure and distribution; (4) PD and clinical outcomes; and (5) potential model‐informed applications. Solid forward arrows indicate the principal treatment–exposure–response sequence represented across the reviewed models. The dashed arrow denotes model‐dependent modifier relationships with PD or clinical outcomes that may not be fully capture by measured CK. Return arrows represent a conceptual iterative learning process in which model outputs may inform subsequent treatment or product/construct design. These connections summarize evidence across heterogeneous models; no single model represented every relationship. They do not establish clinical causality, prospective predictive validation, or regulatory qualification. (B) Categorized dose‐relevant insights. Study‐specific findings are synthesized into three potentially overlapping application categories: product/construct design, dose and regimen, and delivery and translation. Statements reflect study‐specific exploratory findings and should not be interpreted as rankings, universally applicable conclusions, or prospective clinical validation. AUC, area under the cellular concentration–time curve; CAR, chimeric antigen receptor; CAR+, CAR positive; CAR‐T, chimeric antigen receptor T‐cell therapy; CK, cellular kinetics; *C*_max_, maximum measured or model‐predicted CAR‐T cell level; CRS, cytokine release syndrome; E–R, exposure–response; ICANS, immune effector cell‐associated neurotoxicity syndrome; mRNA, messenger RNA; PD, pharmacodynamics; PFS, progression‐free survival; *T*_max_, time to *C*_max_.

Forward arrows in the map show the principal conceptual sequence represented across the reviewed models. The dashed pathway indicates that some modifiers may influence PD or clinical outcomes through relationships not fully captured by measured CK. The return pathway represents iterative use of model outputs to inform subsequent treatment, study, and product or construct design.

### 3.5 Regimen and Treatment‐Strategy Applications

Figure 3 summarizes the model‐informed applications identified across the review. Regimen optimization was assigned only when a modifiable treatment feature such as lymphodepletion, administration route, schedule, fractionation, redosing, or treatment sequencing was directly compared. Product or construct optimization and toxicity‐risk modeling were classified separately as treatment‐strategy applications. The applications were generally exploratory or hypothesis‐generating. No included study prospectively evaluated a clinical dose or regimen recommendation using an independent clinical dataset.

Lymphodepletion and immune reconstitution were recurring regimen considerations. Using a literature‐informed theoretical model, Owens and Bozic (2021) compared chemotherapy schedules, the interval between chemotherapy and CAR‐T infusion, and CAR‐T dose levels. Schedules using the same total chemotherapy amount and CAR‐T dose produced different simulated outcomes [50]. Derippe et al. [51] modeled the relationships among fludarabine/cyclophosphamide with or without alemtuzumab, host lymphocytes, IL‐7, and allogeneic UCART19 CK. The model predicted that alemtuzumab‐containing lymphodepletion could promote UCART19 expansion by increasing IL‐7 availability and reducing host T cell‐mediated elimination; clinical response was not directly modeled [51]. Kimmel et al. [48] simulated a second CAR‐T infusion with and without renewed lymphodepletion. Renewed lymphodepletion was predicted to be necessary within the model to restore CAR‐T expansion [48]. Parmar et al. [58] compared clinical solid‐tumor cohorts with and without prior lymphodepletion and quantified its modeled effects on host‐cell competition, CAR‐T expansion, and persistence.

Administration route and treatment sequencing were evaluated mainly in preclinical solid‐tumor models. Tsai et al. [53] compared intrapleural with intravenous administration and hepatic‐artery with portal‐vein and intravenous administration using mouse minimal PBPK/PD models. In liver‐tumor simulations, hepatic‐artery delivery increased initial tumor infiltration and accelerated early CAR‐T proliferation, whereas portal‐vein delivery provided little predicted advantage over intravenous administration [53]. Hadjigeorgiou et al. [57] developed a PBPK model informed by mouse GBM data to compare local and systemic delivery, anti‐VEGF treatment followed by CAR‐T therapy, timing of a second CAR‐T infusion, and combined vascular and stromal normalization. The model predicted that vascular normalization could improve tumor infiltration and reduce the CAR‐T dose required for tumor control by approximately 5‐fold [57]. Singh et al. [33] also compared intravenous with intratumoral administration in preclinical models [33]. These findings were not established as clinical dose reductions, treatment sequences, or delivery recommendations.

Schedule, fractionation, and repeat administration were examined in several models. León‐Triana et al. [46] proposed repeat CAR‐T administration as a theoretical strategy for controlling CD19‐positive relapse. Barros et al. [47] compared single and fractionated dosing in preclinical hematologic tumor models and explored tumor rechallenge and combination treatment with the indoleamine 2,3‐dioxygenase inhibitor 1‐methyltryptophan [47]. Singh et al. [33] compared single and multiple dosing regimens in preclinical xenograft models [33]. Yang et al. [60] compared single‐infusion, equal‐fraction, and step‐up schedules in virtual patients and predicted similar efficacy among schedules delivering the same total dose. Although the platform was evaluated using held‐out experimental data, these clinical regimen projections were not prospectively evaluated in an independent clinical dataset [60]. For in vivo CAR‐T generation, Kim et al. [55] simulated different mRNA‐loaded polymeric nanoparticle doses and administration intervals to sustain transient CAR expression; no human evaluation was performed [55]. Singh et al. [34] proposed multiple‐infusion simulations as a future application, but its reported analysis evaluated only single‐infusion dose levels and was therefore not classified as a regimen comparison [34].

Product and construct design represented distinct treatment‐strategy applications. Prybutok et al. [52] explored a design space comprising CAR‐T dose, CD4 ratio, CAR affinity, and tumor‐ and healthy‐cell antigen density without comparing alternative schedules [52]. Salem et al. [54] modeled subset‐ and phenotype‐specific CK, including differences between CAR‐positive CD4 and CD8 cells and longitudinal immunophenotypic transitions. Singh et al. [33] linked CAR affinity and density, antigen abundance, effector‐to‐target ratio, cellular expansion, cytokine release, and tumor‐cell killing [33]. Kara et al. [56] explored cyclophosphamide‐associated and enhanced bystander killing as adjunct strategies but did not optimize a specific drug regimen. These analyses generated product, construct, or combination‐treatment hypotheses but did not establish validated manufacturing specifications or clinical regimens.

Toxicity‐focused models primarily supported risk characterization and mitigation hypotheses. Hardiansyah and Ng [32] simulated relationships among CAR‐T dose, baseline tumor burden, CK, B‐cell depletion, and inflammatory cytokines. The model was fitted principally to data from two patients with CLL, and external comparisons used published aggregate metrics rather than independent patient‐level data [32]. Santurio et al. [59] modeled CAR‐T–tumor dynamics, macrophage activation, and IL‐6 kinetics and generated hypotheses concerning tumor debulking and inhibition of macrophage activation. The study did not optimize a specific intervention or schedule [59]. Stein et al. [21] found no detectable association between observed tocilizumab or corticosteroid treatment and tisagenlecleucel expansion rate, but alternative supportive‐care doses or schedules were not compared. These analyses informed toxicity‐risk characterization or mitigation hypotheses rather than demonstrating optimized toxicity‐management regimens.

### 3.6 Biological Process Coverage

Biological process coverage across the 19 reviewed studies was uneven. Based on the review extraction, CAR‐T expansion was represented across all the reviewed studies, consistent with its central role in living‐cell pharmacology. Other CK processes were less consistently represented, including contraction or elimination, memory or persistence, phenotype transition, trafficking or biodistribution, and exhaustion [21, 53, 54, 57, 58, 60].

Tumor response was also frequently modeled, particularly tumor growth and CAR‐T‐mediated killing. More specialized tumor‐related processes, including antigen heterogeneity or escape, resistant‐clone dynamics, bystander killing, and tumor‐site constraints, appeared in a smaller subset of studies [33, 46, 47, 56, 59, 60]. Host immune processes were represented more selectively, including lymphodepletion, endogenous immune reconstitution, IL‐7 dynamics, cytokine release, macrophage‐mediated inflammatory signaling, and T cell competition [32, 48, 50, 51, 58, 59]. Clinical outcome or safety components were less frequent than CK or tumor‐killing components; selected models incorporated CRS, B‐cell aplasia, relapse, survival, or progression‐free survival, whereas ICANS was rarely or not explicitly modeled [21, 32, 34, 46, 49, 59].

### 3.7 Model Evaluation Approaches

Reported model evaluation approaches varied substantially across the reviewed studies (Table 2). Evaluation was often commensurate with exploratory or translational aims, but no study reported prospective external clinical validation using an independent dataset collected to test a prespecified dose‐selection or regimen‐optimization prediction. Based on reviewer assessment, nine studies were judged to have fitted, calibrated, or empirically anchored their models using clinical, patient‐level, or clinical literature‐informed data, with internal consistency assessed against the data used for model development or fitting [21, 32, 34, 49, 51, 54, 57, 58, 59]. Six studies utilized preclinical, in vitro/in vivo, or literature‐derived data for calibration or benchmarking without direct patient‐level clinical fitting [33, 47, 53, 55, 56, 60]. Four studies were primarily theoretical or simulation‐based, with limited indirect benchmarking [46, 48, 50, 52].

### 3.8 Model Credibility and Reporting Appraisal

An adapted PROBAST‐inspired framework was used to appraise credibility and reporting features relevant to mechanistic and semi‐mechanistic CAR‐T models in oncology. Because PROBAST was initially developed for prediction‐model studies rather than mechanistic pharmacological models, the ratings were interpreted as reviewer judgments about credibility support and reporting completeness, not as formal PROBAST risk‐of‐bias judgments. The appraisal considered data relevance, biological plausibility of model inputs and assumptions, endpoint definition, empirical anchoring, sensitivity or uncertainty analysis, and model evaluation. Figure 2B summarizes the study‐level results.

Five studies were judged to have lower concern because they included stronger empirical anchoring, transparent assumptions, sensitivity or uncertainty analyses, and some form of internal model evaluation [21, 49, 51, 58, 59]. Five studies were judged to have higher concern, mainly because evaluation relied on simulation‐only assessment, limited empirical anchoring, or substantial extrapolation from preclinical or theoretical systems. Nine studies were classified as unclear because evaluation was partial, reporting was incomplete, or assessment against external clinical data was unavailable.

Overall, the appraisal provides a structured indication of how far each model's conclusions can reasonably be extended toward CAR‐T dose‐relevant decision making or regimen optimization.

## 4 Discussion

### 4.1 Principal Findings and Comparison With Previous Literature

This systematic scoping review identified 19 studies reporting CAR‐T models classified as mechanistic or semi‐mechanistic models relevant to dose or regimen decision making in oncology. The studies comprised 13 reviewer‐classified mechanistic and six semi‐mechanistic models spanning CK/PD, QSP, PBPK/PD, population CK, stochastic, and agent‐based approaches (Figure 2A). Fifteen studies directly evaluated or varied an administered cell or vector dose or dose range, whereas four addressed dose relevance indirectly through determinants of cellular exposure or response.

The principal finding was that nominal administered dose provides the initial treatment input but does not independently determine subsequent CAR‐T exposure or response. The models identified product, patient, disease, and treatment‐context factors that could modify expansion, persistence, tumor response, or toxicity. Applications included evaluation of dose levels, schedules, fractionation, redosing, lymphodepletion, administration route, product or construct attributes, and risk‐mitigation strategies. However, no study prospectively evaluated a clinical dose or regimen recommendation in an independent clinical dataset. Previous reviews have addressed related areas. Chaudhury et al. [36] provided a narrative overview and mathematical taxonomy of CAR‐T CK and PD modeling, whereas Murias‐Closas et al. [31] reviewed computational CAR‐T models more broadly from CK to patient‐level prediction. The present review extends the literature by focusing on dose‐relevant exposure–response interpretation and regimen or treatment‐strategy applications, and distinguishing exploratory applications from the model evaluation required to support a proposed clinical or regulatory use.

Minucci et al. [61] was treated as an eligibility‐boundary case. Although relevant to individualized CK/PD prediction, it was excluded from the primary synthesis because it did not explicitly evaluate dose levels, dose ranges, dose schedules, or regimen‐optimization scenarios.

### 4.2 Interpretation of Findings

The relationship between nominal dose and in vivo cellular exposure varied across models, products, and disease settings. Stein et al. [21] detected no relationship between tisagenlecleucel dose and *C*_max_ or other model parameters, and León‐Triana et al. [46] predicted a limited dose effect under antigen‐rich conditions. Conversely, Singh et al. [33, 34] predicted steep and eventually plateauing dose‐exposure–response relationships, while other models showed dose‐dependent behavior within their specific experimental or simulated ranges [47, 50, 55, 59, 60]. These findings do not establish that nominal dose is unimportant or that non‐dose determinants are consistently more influential. Rather, the apparent dose effect depends on the product, indication, evaluated dose range, and biological processes represented.

The conceptual framework in Figure 3 illustrates this context dependence. Administered treatment inputs are linked to cellular exposure through product‐, patient‐, disease‐, and treatment‐context factors. Cellular expansion, contraction, persistence, and distribution are then linked to PD and clinical outcomes. Because CAR‐T cells proliferate, differentiate, traffic, and decline after administration, the infused cell number does not prescribe the subsequent exposure profile. Peripheral blood measurements may also incompletely represent exposure at the tumor site, particularly in solid tumors.

The reviewed models further showed that dose‐relevant questions extend beyond selecting a nominal dose. Lymphodepletion and host immune reconstitution influenced modeled expansion and persistence [48, 51, 58], whereas administration route and tumor access affected predicted local exposure [53, 57]. Several studies compared fractionation, redosing, treatment sequencing, or dosing intervals [33, 46, 47, 48, 50, 55, 57, 60]. Product or construct characteristics including CAR affinity, receptor density, CD4 composition, phenotype, and potency, were also linked to CK or response [33, 49, 52, 54, 60]. These product‐design applications can inform treatment development but should remain distinct from regimen optimization.

Overall, the evidence supports an integrated dose‐exposure‐response framework rather than interpretation based on nominal dose alone. The reported relationships remain conditional on each model's data, structure, and assumptions and should not be treated as universal dosing rules.

### 4.3 Clinical, Translational, and Regulatory Significance

The reviewed models primarily demonstrated translational utility by integrating biological, experimental, and clinical information to explain CAR‐T behavior, generate hypotheses, and compare treatment strategies. Their most defensible current applications are mechanistic interpretation, retrospective exposure–response analysis, biomarker prioritization, trial‐design support, and comparison of plausible dose or regimen scenarios. These uses can be valuable even when a model has not been evaluated sufficiently to determine a clinical dose.

Regulatory utility requires a more explicitly decision‐focused context of use. This would specify the question of interest, the model's role and scope, the data and additional evidence contributing to the analysis, and the decision to be informed. Model influence as the intended weight of model outputs relative to other evidence should be considered together with the consequences of an incorrect decision. These elements determine model risk and the extent of evaluation required.

Explicitly defining context of use and model influence would improve the clarity and potential decision relevance of future models by aligning model structure, data generation, uncertainty assessment, and evaluation with the intended application. It would not, by itself, improve predictive performance or establish regulatory acceptability. A model used to explore trial scenarios would generally contribute less weight to a decision than one used as primary evidence for clinical dose selection. The latter application would require correspondingly stronger verification, validation, applicability assessment, and uncertainty characterization.

ICH M15, finalized after the review period, provides a useful prospective framework for these considerations but should not be applied as a retrospective compliance standard. Some of the models in the reviewed studies used clinical data, external aggregate comparisons, or held‐out experimental data. Nevertheless, clinical calibration or experimental evaluation does not establish predictive performance in an independent clinical population. Current evidence is therefore best regarded as exploratory or hypothesis‐generating rather than sufficient for high‐influence regulatory dose or regimen decisions.

Model credibility was also not determined by model class or biological complexity. Some semi‐mechanistic or population models were closely anchored to clinical observations, whereas several detailed mechanistic models relied mainly on in vitro, animal, literature‐derived, or theoretical data. Simplifications of phenotype heterogeneity, antigen evolution, tissue trafficking, tumor microenvironmental barriers, or toxicity pathways may be appropriate for a defined question. However, they constrain extrapolation across products, indications, populations, and regimens. Credibility should therefore be judged in relation to the context of use and supporting evidence, not complexity alone.

### 4.4 Translational Extensions for In Vivo CAR‐T Platforms

In vivo CAR‐T platforms change the definitions of dose and exposure. Ex vivo products administer a measured number of engineered cells, whereas in vivo approaches administer a viral or nonviral vector carrying a genetic payload intended to generate CAR‐expressing immune cells within the patient. For an mRNA‐based platform, the administered dose is therefore the amount of vector or mRNA rather than the number of CAR‐T cells. The resulting exposure pathway may extend from vector disposition and target‐cell uptake through intracellular mRNA release, stability, and translation to the magnitude and duration of CAR expression and the number, phenotype, and distribution of generated CAR‐positive cells. This multistep relationship makes administered vector dose an indirect measure of functional cellular exposure.

Among the reviewed studies, Kim et al. (2024) was the only study that directly modeled an in vivo CAR‐T platform. The authors developed a cell‐level PK/PD model of CAR mRNA delivered by an anti‐CD8‐targeted polymeric nanoparticle. The model linked nanoparticle binding and internalization, intracellular mRNA degradation and translation, transient surface CAR expression, and repeat dosing. Simulations evaluated different mRNA doses and administration intervals and proposed a regimen that sustained modeled CAR expression. Sensitivity analyses identified intracellular mRNA and surface‐protein stability as important determinants of expression [55]. However, the in vivo model was developed using preclinical data from a single experimental dose level, and the simulated regimen was not independently evaluated or translated to humans. The model was proposed as a potential cellular module for future integration into a PBPK framework rather than as a completed preclinical‐to‐clinical translation.

Mechanisms represented in ex vivo CAR‐T models including antigen density, target‐cell burden, host immune status, tissue trafficking, target engagement, and cytokine‐related toxicity may inform downstream modeling of the generated CAR‐positive cells. Their structures and parameter values cannot, however, be assumed to transfer directly to in vivo platforms. Future models will need to couple vector disposition, target‐cell transfection, CAR‐expression kinetics, generated‐cell CK, and downstream efficacy and safety. Platform‐specific considerations may also include off‐target transduction, innate responses to the vector or nanoparticle, repeat‐dose immunogenicity, and the potentially different role of lymphodepletion. Until supported by multidose and human data, these applications should be considered hypothesis‐generating rather than validated tools for clinical dose or regimen selection.

### 4.5 Strength and Limitations

A principal strength of this review was its specific focus on dose‐relevant decision making rather than CAR‐T modeling in general. The inclusion of diverse frameworks enabled comparison across CK/PD, QSP, PBPK/PD, population, stochastic, agent‐based, and in vivo vector models. Explicit definitions distinguished direct from indirect dose relevance, regimen comparison from product or construct optimization, and calibration from independent evaluation. Figures 2, 3, 4 integrate these findings across model structure, exposure–response interpretation, applications, credibility, and toxicity‐modeling coverage.

**FIGURE 4:**
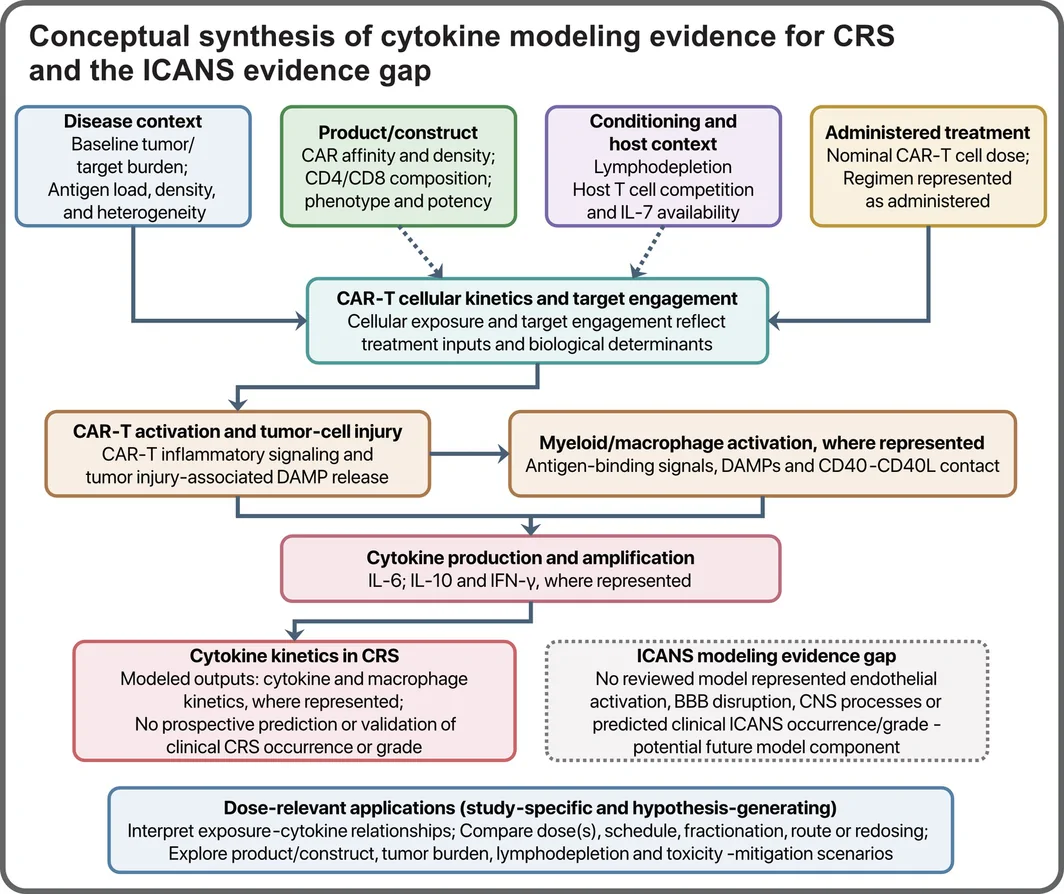
Conceptual synthesis of CRS‐related cytokine modeling and the ICANS mechanistic evidence gap in CAR‐T cell therapy. Cross‐study synthesis of biological and treatment‐related processes represented in mechanistic and semi‐mechanistic models addressing CAR‐T CK, cytokine dynamics, and CRS. Solid arrows denote CRS‐ or cytokine‐related relationships. Dashed arrows denote indirect CK linkages, but not evaluated as CRS pathways. The absence of an arrow to the dashed ICANS box indicated that no ICANS pathway was represented in the reviewed studies; ICANS is shown only as a potential future model component. Applications are study‐specific and hypothesis‐generating; no single model included all components. BBB, blood–brain barrier; CAR, chimeric antigen receptor; CAR‑T, chimeric antigen receptor T‐cell therapy; CD4/CD8, CD4‐positive and CD8‐positive T‐cell subsets; CD40–CD40L, CD40–CD40 ligand signaling; CK, cellular kinetics; CNS, central nervous system; CRS, cytokine release syndrome; DAMP, damage‐associated molecular pattern; ICANS, immune effector cell‐associated neurotoxicity syndrome; IFN‐γ, interferon gamma; IL, interleukin.

Several limitations should be acknowledged. The protocol was not prospectively registered, and the search was limited to eligible peer‐reviewed publications available through December 2025 [62]. Models reported only in regulatory submissions, preprints, conference abstracts, dissertations, proprietary documents, or unpublished analyses may have been missed.

Classification as mechanistic or semi‐mechanistic was reviewer assigned and represents a pragmatic structural distinction rather than a formal ICH category. The adapted PROBAST‐inspired appraisal has not been validated for mechanistic pharmacological models and should be interpreted as a structured assessment of credibility and reporting, not a definitive risk‐of‐bias judgment.

Heterogeneity in products, indications, model structures, data sources, and endpoints precluded quantitative pooling. The synthesis therefore identifies patterns rather than pooled dose effects or comparative model performance. Our appraisal was limited to information available in published articles and supplementary materials; underlying datasets, source code, and computational implementations were not independently verified or reproduced. Finally, the exclusion of nonseparable multi‐antigen products enhanced comparability across studies but reduced representation of emerging platforms, including tandem, bicistronic, pooled, and combination approaches [63].

### 4.6 Future Research Directions

These recommendations address three recurring limitations identified across the reviewed studies: scarcity of mechanistically informative longitudinal clinical data, the trade‐off between biological complexity and parameter identifiability, and the infrequent use of independent data for model evaluation.

Longitudinal measurements of CAR‐T cell phenotype and functional state, tumor antigen expression, tissue distribution, host immune reconstitution, and cytokine dynamics could strengthen mechanistic interpretation but are rarely collected comprehensively in clinical studies. Serial tumor biopsies are invasive, and immunophenotyping, antigen‐expression assays, and tissue‐based measurements are not consistently standardized across investigations. Consequently, peripheral blood CK often remains the primary data source even when tumor‐site processes are central to the biological question.

Mechanistic models must balance biological realism against structural and practical identifiability. Incorporating additional cellular states, signaling pathways, or tissue compartments without corresponding observations can introduce parameters that cannot be estimated uniquely or with sufficient precision. When mechanistic support is limited, simpler model structures, externally informed parameter distributions, or bounded scenario analyses may provide more defensible approaches.

Integrating evidence from multiple sources offers a practical alternative to reliance on a single dataset. Clinical CK/PD data could anchor overall exposure–response relationships; product immunophenotyping and in vitro functional studies can inform cellular subset behavior and potency; preclinical biodistribution studies can inform trafficking processes; and single‐cell RNA sequencing can identify relevant cellular states and candidate pathways. However, single‐cell data do not directly estimate in vivo kinetic parameters, and quantitative translation of in vitro or animal findings to patients may be uncertain.

Accordingly, multisource information may be most appropriately incorporated through linked model components, parameter constraints, or prior distributions rather than pooled without qualification. Differences among products, assays, species, tissues, and disease settings should be considered explicitly, and uncertainty arising from cross‐study or cross‐species translation should be propagated to model predictions.

Future model evaluation would benefit from clearer distinction among calibration, internal assessment, and evaluation using independent data. Relevant activities include verification of model equations and implementation code, assessment of structural and practical identifiability, diagnostic evaluation, sensitivity and uncertainty analyses, and assessment of fitness for the intended product, population, and treatment regimen. As model influence on decision making increases, greater emphasis should be placed on evaluation against independent clinical datasets. Reporting should include model equations, parameter sources, estimation methods, simulation assumptions, software settings, and uncertainty analyses. Reproducible code and model files should be provided where feasible.

Figure 4 highlights an important gap in toxicity modeling. The reviewed CRS models represented selected relationships among dose, disease burden, CAR‐T kinetics, target‐cell injury, inflammatory cytokines, and macrophage activation [32, 33, 59]. Stein et al. [21] examined whether the observed administration of tocilizumab or corticosteroids affected CAR‐T expansion, but the model was not designed to optimize supportive‐care strategies. No included model prospectively predicted clinical CRS grade, and none explicitly modeled the occurrence or severity of ICANS.

Future CRS models could link treatment, product, disease, conditioning, and host factors to CAR‐T kinetics, tumor‐cell injury, myeloid‐cell or macrophage activation, and cytokine trajectories. Extensions for ICANS could incorporate mechanistic hypotheses related to endothelial activation, blood–brain barrier permeability, central nervous system exposure, and neuroinflammation. However, systemic cytokine concentrations or CRS severity should not be assumed to represent validated surrogate markers of ICANS. Progress in this area will likely require time‐aligned biomarker measurements, neurological assessments, standardized clinical endpoints, and detailed treatment‐timing data. Initial applications may be most appropriate for mechanistic exploration, sampling optimization, and comparison of mitigation strategies, whereas more consequential applications would require product‐specific calibration and prospective evaluation using independent clinical data.

In vivo CAR‐T platforms represent an additional opportunity for model development. Kim et al. reported the only included model specifically addressing in vivo CAR‐T generation [55]. Future models could integrate vector biodistribution, cellular uptake, intracellular payload disposition, CAR expression, kinetics of newly generated CAR‐positive cells, target engagement, efficacy, and toxicity. For repeat‐administration strategies, models may also need to represent off‐target delivery, innate immune activation, vector‐specific immune responses, and changes in transfection efficiency over time. Although concepts developed for ex vivo CAR‐T products may provide useful starting points, quantitative translation will require platform‐specific data.

Overall, the literature supports moving beyond interpretation based solely on nominal dose without diminishing the importance of dose itself. CAR‐T exposure and response emerge from interactions among administered dose, product attributes, tumor and antigen characteristics, conditioning regimens, host immune context, and access to disease sites. At present, these models are most strongly supported for mechanistic interpretation, scenario exploration, and hypothesis generation for trial design. Broader application to clinical or regulatory dose and regimen decisions will likely require clearly defined contexts of use, richer longitudinal datasets, transparent integration of multisource evidence, decision‐proportionate evaluation frameworks, and independent clinical assessment.

## Author Contributions

D.O. and G.M. wrote the manuscript; D.O. designed the research; D.O. and G.M. performed the research; D.O. and G.M. analyzed the data.

## Funding

The authors have nothing to report.

## Conflicts of Interest

The authors declare no conflicts of interest.

## Supporting information


**File S1:** Scoping review protocol.

**File S2:** PRISMA‐ScR checklist mapping.

**File S3:** Detailed search strategies for Ovid MEDLINE, Embase, Scopus, and Google Scholar.

**File S4:** Data‐extraction form.

## Data Availability

The data‐extraction form and extracted data supporting the findings of this systematic scoping review are provided with the supplementary materials. All reviewed studies are publicly available publications cited in the References section.
