# Supplementary material for: Application of Mechanistic Modeling to Support Dose‐Relevant Decision Making for CAR‐T Cell Therapy in Oncology: A Systematic Scoping Review
Source: Clin Transl Sci. 2026 Nov 6;19(11):e70756. doi: 10.1111/cts.70756 (PMC13741396; doi:10.1111/cts.70756)
Supplement: Supplementary file 1 [file CTS-19-e70756-s001.docx]

## Supplementary File S2: PRISMA-ScR checklist mapping

**Supplementary Table S2. PRISMA-ScR checklist mapped to planned manuscript sections**

This table maps the 22 PRISMA-ScR checklist items to the corresponding sections of the manuscript and specifies how each item is addressed.

| **PRISMA -ScR item** | **Checklist item** | **Manuscript location** | **How addressed in the manuscript** |
| --- | --- | --- | --- |
| 1 | **Title**: Identify the report as a scoping review. | Title page | The title identifies the article as a scoping review of application of mechanistic modeling to support dose selection and regimen optimization for CAR T cell therapy in oncology. |
| 2 | **Structured summary**: Provide a structured summary including background, objectives, eligibility criteria, sources of evidence, charting methods, results, and conclusions. | Abstract | The abstract summarizes the background, objectives, eligibility criteria, information sources, methods, results, and conclusions. |
| 3 | **Rationale**: Describe the rationale for the review in the context of what is already known and explain why a scoping review approach is appropriate. | Introduction: Clinical context and unmet need; Regulatory context and mechanistic modeling; Review Objectives | The rationale explains why CAR-T dose selection is pharmacologically challenging and why mechanistic modeling may support biologically informed dose and regimen decisions. |
| 4 | **Objectives**: Provide an explicit statement of the questions and objectives. | Introduction: Review Objectives; Supplementary Protocol Sections 2–3 | The review question and objectives are stated, including identification, classification, synthesis, and appraisal of mechanistic and semi-mechanistic CAR-T models relevant to dose selection and regimen optimization in oncology. |
| 5 | **Protocol and registration**: Indicate whether a protocol exists and where it can be accessed; provide registration information if available. | Methods: Protocol and registration; Supplementary Protocol Sections 3–4 | The protocol was developed a priori but was not formally registered in a systematic review registry (such as PROSPERO). The manuscript explains the rationale and describes transparency measures, including pre‑specified eligibility criteria, search strategy, screening process, and data-extraction approach. |
| 6 | **Eligibility criteria**: Specify characteristics of sources of evidence used as criteria for eligibility and provide rationale. | Methods: Eligibility criteria; Supplementary Protocol Sections | Inclusion and exclusion criteria are defined. Eligible studies reported original mechanistic or semi-mechanistic CK/PD, PK/PD, QSP, PBPK/PD, stochastic, or agent-based models applied to CAR-T therapy in oncology and relevant to dose selection, or regimen optimization. |
| 7 | **Information sources**: Describe all information sources and date last searched. | Methods: Search strategy; Supplementary Protocol Section 7; Supplementary Table S1 | Ovid MEDLINE, Embase, and Scopus are listed as primary databases, with Google Scholar used as a supplementary search source. The search period was January 2010 to December 2025. |
| 8 | **Search**: Present the full electronic search strategy for at least one database, including limits. | Methods: Search strategy | The manuscript describes search concepts covering CAR-T therapy, mechanistic modeling, cellular kinetics/pharmacodynamics, and dose or regimen optimization. Full database-specific search strings provided in the supplementary materials. |
| 9 | **Selection of sources of evidence**: State the process for selecting sources of evidence for the review. | Methods: Study selection and data extraction; Supplementary Protocol Section 9 | The manuscript describes title/abstract screening, full-text review, reviewer process, consensus resolution, and the PRISMA flow of included and excluded records. |
| 10 | **Data charting process**: Describe methods of charting data and any process for obtaining or confirming data from investigators. | Methods: Study selection and data extraction; Supplementary Protocol Section 10 | Data extraction was performed using a standardized form and verified by a second reviewer. |
| 11 | **Data items**: List and define all variables for which data will be sought and any assumptions or simplifications that will be made. | Methods: Study selection and data extraction; Model classification framework | Extracted variables included author, year, model class, mathematical framework, indication, target antigen, CAR-T platform, dose-selection relevance, regimen-optimization relevance, biological processes represented, and model-evaluation approach. |
| 12 | **Critical appraisal of individual sources of evidence**: If done, provide rationale and methods. | Methods: Model credibility and reporting appraisal | Because conventional risk-of-bias tools are not designed for mechanistic pharmacological models, the manuscript does not present a conventional risk-of-bias assessment. Instead, it describes an adapted model-credibility and reporting appraisal informed by PROBAST domains, ICH M15 principles, and established CK/PD, QSP, and PBPK model-evaluation concepts. |
| 13 | **Synthesis of results**: Describe methods of handling and summarizing charted data. | Methods: Data synthesis / Model classification framework | The manuscript describes narrative, tabular, and conceptual synthesis by model class, mathematical framework, biological process coverage, dose-selection strategy, regimen-optimization strategy, model evaluation, and credibility considerations. |
| 14 | **Selection of sources of evidence**: Give numbers of sources screened, assessed for eligibility, and included, with reasons for exclusions at each stage. | Results: Study selection; PRISMA flow diagram | The PRISMA flow reports records identified, duplicates removed, records screened, full texts assessed, full-text exclusions, and final included studies. |
| 15 | **Characteristics of sources of evidence**: Present characteristics of included sources. | Results: Study characteristics; Table 2 | Table 2 summarizes study characteristics, including model type, framework, indication, target/product context, evidence level, dose-selection relevance, regimen-optimization relevance, and model evaluation. |
| 16 | **Critical appraisal within sources of evidence**: If done, present data on critical appraisal of included sources. | Results: Model credibility and reporting appraisal; Figure panel on model evaluation/credibility | The manuscript reports credibility and reporting-appraisal findings across included studies, including limitations in context-of-use definition, validation, uncertainty quantification, applicability, and prospective decision support. Findings should be interpreted as a structured appraisal of model credibility and reporting quality, not as a validated risk-of-bias rating for mechanistic models. |
| 17 | **Results of individual sources of evidence**: Present relevant data charted from each source. | Results: Model characteristics and applications; Table 2 | Study-level findings are presented for each included model, including the dose-selection or regimen-optimization concept addressed and the corresponding model-evaluation approach. |
| 18 | **Synthesis of results**: Summarize or present charting results as they relate to the review questions and objectives. | Results: Model types and mathematical frameworks; Biological process coverage; Dose-selection and regimen-optimization applications; Model evaluation approaches | Findings are synthesized across modeling frameworks, biological processes, dose-selection strategies, regimen-optimization applications, CK/PD parameters, model-evaluation approaches, and conceptual study interrelationships. |
| 19 | **Summary of evidence**: Summarize main results and link them to the review questions and objectives. | Discussion: Principal findings / Summary of evidence | The Discussion summarizes the principal findings, including the limited predictiveness of administered cell dose alone, the importance of tumor burden, antigen engagement, product and host factors, the relevance of delivery and trafficking in solid tumors, and the need for stronger model credibility. |
| 20 | **Limitations**: Discuss limitations of the scoping review process. | Discussion: Limitations | The manuscript discusses limitations including lack of prospective protocol registration, reliance on peer-reviewed published literature, subjectivity of M/SM classification, use of an adapted appraisal framework, heterogeneity of model types and indications, and lack of independent verification of model code or datasets. |
| 21 | **Conclusions**: Provide a general interpretation of results and implications for future research and practice. | Discussion: Conclusions and implications for model-informed CAR-T development | The conclusion summarizes the value and current limitations of mechanistic modeling for CAR-T dose selection and regimen optimization in oncology and identifies requirements for more decision-influential regulatory use. |
| 22 | **Funding**: Describe sources of funding for included sources of evidence and for the review, and the role of funders. | Funding statement / acknowledgements | No specific funding was received for this work. |

**Note:** PRISMA-ScR, Preferred Reporting Items for Systematic Reviews and Meta-Analyses extension for Scoping Reviews; CK/PD, cellular kinetic/pharmacodynamic; PK/PD, pharmacokinetic/pharmacodynamic; QSP, quantitative systems pharmacology; PBPK/PD, physiologically based pharmacokinetic/pharmacodynamic; ICH M15, International Council for Harmonisation M15 guideline on model-informed drug development.
Because no validated risk-of-bias tool exists specifically for mechanistic CAR-T modeling studies, Items 12 and 16 are addressed using a structured model-credibility and reporting appraisal rather than a conventional risk-of-bias assessment.
