# Supplementary material for: Application of Mechanistic Modeling to Support Dose‐Relevant Decision Making for CAR‐T Cell Therapy in Oncology: A Systematic Scoping Review
Source: Clin Transl Sci. 2026 Nov 6;19(11):e70756. doi: 10.1111/cts.70756 (PMC13741396; doi:10.1111/cts.70756)
Supplement: Supplementary file 2 [file CTS-19-e70756-s002.docx]

## Supplementary File S3: Detailed search strategies for Ovid MEDLINE, Embase, Scopus, and Google Scholar

**Supplementary Material S3. Full Database Search Strategies**

*Mechanistic model-informed dose selection and regimen optimization strategies for CAR-T cell therapy in oncology*

**Search overview**

| **Element** | **Specification** |
| --- | --- |
| Review type | Systematic scoping review conducted using Joanna Briggs Institute methodology and reported according to PRISMA-ScR. |
| Population / concept / context | CAR-T cell therapy in oncology; original mechanistic or semi-mechanistic CK/PD, PK/PD, QSP, PBPK/PD, stochastic, translational PK/PD, or agent-based models; relevance to dose selection, exposure-response interpretation, or regimen optimization. |
| Date range | January 1, 2010 to December 31, 2025. |
| Primary bibliographic databases | Ovid MEDLINE, Embase, and Scopus |
| Supplementary source | Google Scholar, used for supplementary citation searching and retrieval of recent or cross-disciplinary modeling records. |
| Limits applied | Publication year 2010-2025. No human-only filter was applied because preclinical and translational modeling studies were eligible. Records restricted to English language. |
| Deduplication and screening | Records exported from each database were deduplicated before title/abstract screening. Full texts were assessed against pre‑specified eligibility criteria, including explicit relevance to CAR-T dose selection, exposure-response interpretation, or regimen optimization. |

***Note:*** *Database interfaces can differ in field labels and controlled-vocabulary syntax. The strings below are written for Ovid MEDLINE, Ovid Embase, Scopus, and Google Scholar. If another Embase platform is used, controlled vocabulary and proximity syntax should be translated without changing the conceptual structure of the search.*

**Ovid MEDLINE(R) search strategy ALL <1946 to January 05, 2026>**

| **#** | **Query** | **Results from  5 Jan 2026** |
| --- | --- | --- |
| 1 | exp neoplasms/ | 4,260,337 |
| 2 | cancer*.mp. | 2,767,213 |
| 3 | neoplasm*.mp. | 3,650,948 |
| 4 | 1 or 2 or 3 | 5,190,048 |
| 5 | exp Receptors, Chimeric Antigen/ | 9,072 |
| 6 | exp Immunotherapy, Adoptive/ | 19,508 |
| 7 | 5 and 6 | 7,342 |
| 8 | car t.mp. | 18,703 |
| 9 | cart.mp. | 11,501 |
| 10 | chimeric antigen receptor*.mp. | 17,822 |
| 11 | 7 or 8 or 9 or 10 | 34,651 |
| 12 | exp Models, Biological/ | 992,328 |
| 13 | Mechanistic Model*.mp. | 8,345 |
| 14 | PBPK.mp. | 4,835 |
| 15 | physiologically-based pharmacokinetic.mp. | 5,348 |
| 16 | QSP.mp. | 561 |
| 17 | quantitative systems pharmacology.mp. | 600 |
| 18 | systems pharmacology.mp. | 2,807 |
| 19 | mathematical model*.mp. | 74,170 |
| 20 | computational model*.mp. | 42,143 |
| 21 | "CK/PD model".mp. | 3 |
| 22 | "PK/PD model".mp. | 1,196 |
| 23 | 12 or 13 or 14 or 15 or 16 or 17 or 18 or 19 or 20 or 21 or 22 | 1,084,558 |
| 24 | exp Dose-Response Relationship, Drug/ | 454,465 |
| 25 | dose.mp. | 1,713,578 |
| 26 | dosing.mp. | 117,141 |
| 27 | 24 or 25 or 26 | 1,771,866 |
| 28 | 4 and 11 and 23 and 27 | 63 |
| 29 | limit 28 to dt=20100101-20251231 | 57 |
| 30 | limit 29 to english language | 57 |

**Embase search strategy (Ovid interface)**

| **#** | **Query** | **Results from  5 Jan 2026** |
| --- | --- | --- |
| 1 | exp Neoplasms/ | 4,260,337 |
| 2 | neoplasm*.mp. [mp=title, book title, abstract, original title, name of substance word, subject heading word, floating sub-heading word, keyword heading word, organism supplementary concept word, protocol supplementary concept word, rare disease supplementary concept word, unique identifier, synonyms, population supplementary concept word, anatomy supplementary concept word] | 3,650,948 |
| 3 | cancer*.mp. [mp=title, book title, abstract, original title, name of substance word, subject heading word, floating sub-heading word, keyword heading word, organism supplementary concept word, protocol supplementary concept word, rare disease supplementary concept word, unique identifier, synonyms, population supplementary concept word, anatomy supplementary concept word] | 2,767,213 |
| 4 | tumo?r*.mp. [mp=title, book title, abstract, original title, name of substance word, subject heading word, floating sub-heading word, keyword heading word, organism supplementary concept word, protocol supplementary concept word, rare disease supplementary concept word, unique identifier, synonyms, population supplementary concept word, anatomy supplementary concept word] | 2,899,028 |
| 5 | malignanc*.mp. [mp=title, book title, abstract, original title, name of substance word, subject heading word, floating sub-heading word, keyword heading word, organism supplementary concept word, protocol supplementary concept word, rare disease supplementary concept word, unique identifier, synonyms, population supplementary concept word, anatomy supplementary concept word] | 381,467 |
| 6 | 1 or 2 or 3 or 4 or 5 | 5,887,786 |
| 7 | exp Receptors, Chimeric Antigen/ | 9,072 |
| 8 | chimeric antigen receptor* T cell*.mp. [mp=title, book title, abstract, original title, name of substance word, subject heading word, floating sub-heading word, keyword heading word, organism supplementary concept word, protocol supplementary concept word, rare disease supplementary concept word, unique identifier, synonyms, population supplementary concept word, anatomy supplementary concept word] | 6,884 |
| 9 | CAR T.mp. [mp=title, book title, abstract, original title, name of substance word, subject heading word, floating sub-heading word, keyword heading word, organism supplementary concept word, protocol supplementary concept word, rare disease supplementary concept word, unique identifier, synonyms, population supplementary concept word, anatomy supplementary concept word] | 18,703 |
| 10 | 7 or 8 or 9 | 21,894 |
| 11 | Computer Simulation/ | 232,103 |
| 12 | (computer* adj3 simulat*).mp. [mp=title, book title, abstract, original title, name of substance word, subject heading word, floating sub-heading word, keyword heading word, organism supplementary concept word, protocol supplementary concept word, rare disease supplementary concept word, unique identifier, synonyms, population supplementary concept word, anatomy supplementary concept word] | 252,772 |
| 13 | (mechanistic adj3 (model* or silico)).mp. [mp=title, book title, abstract, original title, name of substance word, subject heading word, floating sub-heading word, keyword heading word, organism supplementary concept word, protocol supplementary concept word, rare disease supplementary concept word, unique identifier, synonyms, population supplementary concept word, anatomy supplementary concept word] | 13,827 |
| 14 | (mathematical adj3 model*).mp. [mp=title, book title, abstract, original title, name of substance word, subject heading word, floating sub-heading word, keyword heading word, organism supplementary concept word, protocol supplementary concept word, rare disease supplementary concept word, unique identifier, synonyms, population supplementary concept word, anatomy supplementary concept word] | 78,234 |
| 15 | PBPK.mp. [mp=title, book title, abstract, original title, name of substance word, subject heading word, floating sub-heading word, keyword heading word, organism supplementary concept word, protocol supplementary concept word, rare disease supplementary concept word, unique identifier, synonyms, population supplementary concept word, anatomy supplementary concept word] | 4,835 |
| 16 | physiologically-based pharmacokinetic.mp. [mp=title, book title, abstract, original title, name of substance word, subject heading word, floating sub-heading word, keyword heading word, organism supplementary concept word, protocol supplementary concept word, rare disease supplementary concept word, unique identifier, synonyms, population supplementary concept word, anatomy supplementary concept word] | 5,348 |
| 17 | QSP.mp. [mp=title, book title, abstract, original title, name of substance word, subject heading word, floating sub-heading word, keyword heading word, organism supplementary concept word, protocol supplementary concept word, rare disease supplementary concept word, unique identifier, synonyms, population supplementary concept word, anatomy supplementary concept word] | 561 |
| 18 | quantitative systems pharmacology.mp. [mp=title, book title, abstract, original title, name of substance word, subject heading word, floating sub-heading word, keyword heading word, organism supplementary concept word, protocol supplementary concept word, rare disease supplementary concept word, unique identifier, synonyms, population supplementary concept word, anatomy supplementary concept word] | 600 |
| 19 | 11 or 12 or 13 or 14 or 15 or 16 or 17 or 18 | 338,255 |
| 20 | 6 and 10 and 19 | 125 |
| 21 | limit 20 to english language | 125 |

**Scopus search strategy**

TITLE-ABS-KEY(
("CAR T" OR "CAR-T" OR "chimeric antigen receptor*" OR "chimeric antigen receptor T cell*" OR "chimeric antigen receptor T-cell*")
AND (cancer* OR tumor* OR tumour* OR neoplas* OR malignan* OR oncology OR lymphoma* OR leukemia* OR leukaemia* OR myeloma*)
AND (model* OR simulation* OR "cellular kinetic*" OR pharmacokinetic* OR pharmacodynamic* OR "PK/PD" OR "CK/PD" OR "quantitative systems pharmacology" OR QSP OR PBPK OR "dose selection" OR "dose optimization" OR "dose optimisation" OR "exposure-response" OR "exposure response" OR "regimen optimization" OR "regimen optimisation"))
AND PUBYEAR > 2010 AND PUBYEAR < 2025

**Google Scholar supplementary search strategy**

| **No.** | **Google Scholar query** |
| --- | --- |
| 1 | "CAR-T" "mechanistic model" dose cancer |
| 2 | "chimeric antigen receptor" "cellular kinetics" dose cancer |
| 3 | "CAR-T" "quantitative systems pharmacology" cancer dose |
| 4 | "CAR-T" QSP "dose" cancer |
| 5 | "CAR-T" PBPK cancer dosing |
| 6 | "CAR-T" "PK/PD" "dose selection" cancer |
| 7 | "CAR-T" "exposure-response" cancer |
| 8 | "CAR-T" "regimen optimization" cancer |
| 9 | "CAR-T" "lymphodepletion" model dose cancer |
| 10 | "CAR-T" "agent-based model" solid tumor cancer |
| 11 | "CAR-T" stochastic model dose cancer |
| 12 | "CAR-T" "mRNA" "dose" "model" cancer |

***Notes:*** *Google Scholar was used as a supplementary source because it is not fully reproducible and does not provide stable exportable search histories. Searches should be run with the custom year range 2010-2025. The first 100-200 results per query, sorted by relevance, may be screened until no additional eligible records are identified. All potentially eligible records should be deduplicated against the primary database exports and assessed by full-text review.*

**Eligibility-relevant search decision rules**

| **Screening issue** | **Decision rule** |
| --- | --- |
| Mechanistic model without oncology application | Exclude as non-cancer indication. |
| CAR-T CK/PD characterization without dose, exposure-response, or regimen-optimization application | Exclude as not dose-related, even if the authors state that the model may support future dose prediction. |
| Preclinical or translational model evaluating dose, schedule, delivery route, lymphodepletion, vector/mRNA dose, or tumor-site exposure | Include if original model development and sufficient methods are reported. |
| Purely statistical or descriptive model without mechanistic biological structure | Exclude as non-mechanistic. |
| Review, editorial, perspective, or regulatory assessment without original model development | Exclude as secondary literature. |
