# Supplementary material for: Application of Mechanistic Modeling to Support Dose‐Relevant Decision Making for CAR‐T Cell Therapy in Oncology: A Systematic Scoping Review
Source: Clin Transl Sci. 2026 Nov 6;19(11):e70756. doi: 10.1111/cts.70756 (PMC13741396; doi:10.1111/cts.70756)
Supplement: Supplementary file 3 [file CTS-19-e70756-s003.docx]

## Supplementary File S4: Data-extraction form

**Supplementary Material S4. Data-Extraction Form**

*Scoping review of mechanistic and semi-mechanistic modeling approaches for CAR-T dose selection and regimen optimization in oncology*

**Purpose:** This form is designed to standardize extraction of study, model, dose-selection, regimen-optimization, biological process, and model-evaluation information from eligible publications. It is intended for use by the primary extractor and independent verifier. Fields marked “not reported” should not be inferred unless directly supported by the source text, tables, figures, or supplementary materials.

**A. Administrative and screening information**

| **Field / variable** | **Permitted values or coding guidance** | **Data to extract** | **Source / notes** |
| --- | --- | --- | --- |
| Study ID | Sequential ID assigned during screening |  | e.g., S001 |
| First author and year | Author surname, year |  | Use citation format: Author et al. (Year) |
| Record source | Ovid MEDLINE / Embase / Scopus / Google Scholar / hand-search / targeted verification |  |  |
| DOI / PMID / URL | Record all available persistent identifiers |  |  |
| Title | Full article title |  |  |
| Journal | Journal name |  |  |
| Publication type | Original research / methods paper / brief report / other |  | Exclude reviews, editorials, perspectives without original model development |
| Peer-reviewed full text available? | Yes / No / unclear |  |  |
| Screening decision | Include / exclude / uncertain |  |  |
| Full-text exclusion reason, if applicable | Not CAR-T; non-cancer; non-mechanistic; not dose-related; review/no original model; insufficient methods; other |  | Use one primary reason only; add details in notes |
| Extractor | Initials |  |  |
| Verifier | Initials |  |  |
| Date extracted / verified | DD-MMM-YYYY |  |  |

**B. Eligibility confirmation**

| **Field / variable** | **Permitted values or coding guidance** | **Data to extract** | **Source / notes** |
| --- | --- | --- | --- |
| Original model development? | Yes / No / unclear |  | Must report original mechanistic or semi-mechanistic model development, adaptation, or calibration |
| CAR-T therapy? | Yes / No / unclear |  | Model must be specific to CAR-T or in vivo CAR generation |
| Oncology indication? | Yes / No / unclear |  | Exclude non-cancer CAR-T applications |
| Dose-selection relevance? | Yes / No / unclear |  | Includes explicit dose-level, dose-range, exposure-response, dose-response, starting-dose, functional-dose, or product-dose question |
| Regimen-optimization relevance? | Yes / No / unclear |  | Includes lymphodepletion, schedule, fractionation, route, repeat dosing, vector/mRNA dose, combination timing, toxicity intervention, or product-composition optimization |
| Exposure-response relevance? | Yes / No / unclear |  | Includes CK/PD, PK/PD, Cmax/AUC, expansion, persistence, tumor response, toxicity, survival, or other outcome linkage |
| Final eligibility decision | Included / excluded after full text / pending adjudication |  | Reviewer consensus required for boundary cases |
| Boundary-case notes | Free text |  | Document rationale for inclusion/exclusion, e.g., CK/PD characterization without explicit dose application |

**C. Study and clinical context**

| **Field / variable** | **Permitted values or coding guidance** | **Data to extract** | **Source / notes** |
| --- | --- | --- | --- |
| CAR-T platform | Autologous ex vivo / allogeneic ex vivo / in vivo CAR-T / conceptual / not product-specific |  |  |
| Product or construct | Named product or investigational construct; not product-specific if unspecified |  | e.g., tisagenlecleucel, UCART19, LB1908, mRNA-LNP generated CAR-T |
| Target antigen(s) | CD19 / BCMA / mesothelin / CEA / claudin18.2 / HER2 / EGFR / FAP / multiple / not reported |  |  |
| Cancer indication | B-ALL / NHL / DLBCL / MM / solid tumor / cancer NOS / other |  |  |
| Disease context | Hematologic malignancy / solid tumor / solid-tumor relevant / cross-context |  |  |
| Development phase represented | Preclinical / translational / clinical retrospective / clinical early phase / theoretical / in silico |  | Use highest level of empirical data used for calibration/evaluation |
| Species / system | Human / mouse / in vitro / virtual patient / mixed / not applicable |  |  |
| Data source used for model building | Original data / published clinical data / published preclinical data / literature parameterization / simulation only |  |  |
| Sample size or dataset size | Number of patients, animals, experiments, virtual patients, or datasets |  | Record as reported; use not reported where absent |
| Clinical endpoint(s) | Response / CR / tumor burden / survival / CRS / ICANS / B-cell aplasia / CK only / other |  |  |

**D. Model classification and framework**

| **Field / variable** | **Permitted values or coding guidance** | **Data to extract** | **Source / notes** |
| --- | --- | --- | --- |
| Model class | Mechanistic (M) / semi-mechanistic (SM) / uncertain |  | Apply pre‑specified study classification rule |
| Classification rationale | Dominant organizing structure and inference basis |  | Explain why M or SM |
| Mathematical framework | ODE / PDE / PBPK / mPBPK / QSP / NLME / stochastic / agent-based / multiscale / other |  |  |
| Software / implementation | NONMEM / MATLAB / SimBiology / R / Python / custom / not reported |  |  |
| Model compartments or agents | Free text |  | List key compartments, cell states, tissues, or agents |
| Scales represented | Molecular / cellular / tissue / organ / whole-body / population / multiscale |  |  |
| Model structure availability | Equations reported / code available / schematic only / insufficient detail |  |  |
| Parameter source | Estimated / fixed from literature / calibrated / assumed / mixed / not reported |  |  |
| Identifiability assessed? | Yes / No / unclear / not applicable |  |  |
| Uncertainty quantified? | None / local sensitivity / global sensitivity / bootstrap / Bayesian / Monte Carlo / confidence intervals / other |  |  |

**E. Dose-selection and regimen-optimization application**

| **Field / variable** | **Permitted values or coding guidance** | **Data to extract** | **Source / notes** |
| --- | --- | --- | --- |
| Dose-selection category | Nominal dose / functional dose / exposure-guided / tumor burden-informed / antigen-informed / product-attribute-informed / vector or mRNA dose / not applicable |  |  |
| Specific dose question | Free text |  | State exactly what dose-related question was addressed |
| Dose levels or ranges evaluated | Free text; not reported if absent |  | Record actual values and units |
| Exposure metric(s) | Cmax / AUC / expansion rate / persistence / CAR expression / tumor-site exposure / transgene copies / other |  |  |
| Dose-response or exposure-response analysis? | Yes / No / partial / unclear |  | Document whether dose/exposure was linked to efficacy or toxicity |
| Regimen-optimization category | Lymphodepletion / schedule / fractionation / repeat dosing / route / local delivery / combination timing / toxicity intervention / product composition / not applicable |  |  |
| Regimen components modeled | Free text |  | e.g., fludarabine/cyclophosphamide, alemtuzumab, anti-VEGF, tocilizumab, mRNA-LNP redosing |
| Route or delivery modeled? | IV / local / intratumoural / intrapleural / hepatic artery / portal vein / vector delivery / not applicable |  |  |
| Prospective clinical decision support? | Yes / No / unclear |  | Indicate whether the model was used prospectively to select a clinical dose or regimen |
| Recommended interpretation | Clinical decision-support / retrospective clinical interpretation / translational hypothesis-generating / preclinical design-space exploration / theoretical |  |  |

**F. Biological processes represented**

| **Field / variable** | **Permitted values or coding guidance** | **Data to extract** | **Source / notes** |
| --- | --- | --- | --- |
| CAR-T expansion | Yes / No / unclear |  |  |
| Contraction or elimination | Yes / No / unclear |  |  |
| Persistence or memory phenotype | Yes / No / unclear |  |  |
| CD4+/CD8+ or cell-subset dynamics | Yes / No / unclear |  |  |
| Exhaustion or dysfunction | Yes / No / unclear |  |  |
| Trafficking or biodistribution | Yes / No / unclear |  |  |
| Tumor growth | Yes / No / unclear |  |  |
| Tumor killing | Yes / No / unclear |  |  |
| Antigen density or target engagement | Yes / No / unclear |  |  |
| Antigen escape or resistant subclones | Yes / No / unclear |  |  |
| Bystander killing | Yes / No / unclear |  |  |
| Lymphodepletion or immune reconstitution | Yes / No / unclear |  |  |
| Cytokines or CRS mediators | Yes / No / unclear |  | e.g., IL-6, IFN-gamma, GM-CSF |
| Macrophage/monocyte activation | Yes / No / unclear |  |  |
| Tumor microenvironment barriers | Yes / No / unclear |  | e.g., hypoxia, vascular normalization, stromal barrier, immunosuppression |
| Manufacturing or product attributes | Yes / No / unclear |  | e.g., viability, phenotype, transduction, CAR affinity |
| Clinical safety endpoints | CRS / ICANS / cytopenia / B-cell aplasia / none / other |  |  |
| Clinical efficacy endpoints | Tumor burden / response / relapse / survival / none / other |  |  |

**G. Key parameters and quantitative outputs**

| **Field / variable** | **Permitted values or coding guidance** | **Data to extract** | **Source / notes** |
| --- | --- | --- | --- |
| Key CK parameters | Expansion rate, doubling time, contraction half-life, persistence half-life, carrying capacity, etc. |  | Record values, units, confidence intervals if reported |
| Key PD parameters | Tumor growth, kill rate, antigen binding, cytokine production, toxicity threshold, etc. |  |  |
| Covariates or predictors | Tumor burden / antigen / product attributes / lymphodepletion / age / disease state / other |  |  |
| Model outputs | CK profiles / tumor burden / response probability / CRS risk / survival / tumor-site exposure / virtual patients / other |  |  |
| Sensitivity findings | Top-ranked influential parameters |  |  |
| Main dose-related finding | Free text |  | Use cautious wording; distinguish findings from implications |
| Main regimen-related finding | Free text |  |  |
| Limitations stated by authors | Free text |  |  |
| Limitations judged by reviewers | Free text |  | Include over-extrapolation, lack of validation, unsupported product-specific claims, etc. |

**H. Model evaluation, credibility, and reporting appraisal**

| **Field / variable** | **Permitted values or coding guidance** | **Data to extract** | **Source / notes** |
| --- | --- | --- | --- |
| Context of use stated? | Clear / partial / absent |  | Specify stated model purpose and decision context |
| Verification reported? | Yes / partial / no / unclear |  | Numerical checks, solver stability, code checks, mass balance, stochastic seed reproducibility |
| Calibration reported? | Clinical / preclinical / in vitro / literature / simulation only / not reported |  |  |
| Internal evaluation reported? | Goodness-of-fit / VPC / residual diagnostics / cross-validation / sensitivity / none / other |  |  |
| External validation reported? | Independent clinical / independent preclinical / none / unclear |  | Reserve “external” for independent data not used in model development |
| Acceptance criteria pre‑specified? | Yes / no / unclear |  |  |
| Applicability domain discussed? | Clear / partial / absent |  | Population, product, indication, dose range, endpoint, and extrapolation limits |
| Credibility concern: data | Low / moderate / high / unclear |  | Appropriateness, sample size, representativeness, clinical relevance |
| Credibility concern: inputs | Low / moderate / high / unclear |  | Biological plausibility, parameter source, identifiability |
| Credibility concern: endpoints | Low / moderate / high / unclear |  | Clinical relevance and endpoint definition |
| Credibility concern: analysis/evaluation | Low / moderate / high / unclear |  | Model evaluation adequacy relative to intended use |
| Overall appraisal | Clinically anchored / translational / preclinical / hypothesis-generating / insufficiently reported |  | This is a model-credibility and reporting appraisal, not a conventional validated risk-of-bias rating |

**I. Reviewer adjudication and synthesis coding**

| **Field / variable** | **Permitted values or coding guidance** | **Data to extract** | **Source / notes** |
| --- | --- | --- | --- |
| Reviewer 1 inclusion decision | Include / exclude / uncertain |  |  |
| Reviewer 2 inclusion decision | Include / exclude / uncertain |  |  |
| Consensus decision | Include / exclude / adjudication needed |  |  |
| Primary synthesis category | Dose selection / regimen optimization / exposure-response interpretation / model-evaluation framework / other |  |  |
| Secondary synthesis category | Free text |  |  |
| Included in Table 1? | Yes / no |  |  |
| Included in biological process table? | Yes / no |  |  |
| Included in conceptual network figure? | Yes / no |  |  |
| Citation for manuscript reference list | Full reference |  | Check accuracy against journal format |
| Final notes | Free text |  |  |

**J. Coding notes and definitions**

| **Term** | **Operational definition / coding rule** |
| --- | --- |
| Mechanistic (M) | Models in which biological mechanisms, system compartments, spatial architecture, or physiologic processes are the dominant organizing structure. |
| Semi-mechanistic (SM) | Models in which a mechanistic core is combined with empirical, population, statistical, or translational components that substantially influence inference or prediction. |
| Dose-related inclusion threshold | The study must explicitly evaluate or interpret dose, exposure-response, dose-response, dose levels/ranges, vector/mRNA dose, starting dose, functional dose, or a regimen component that influences dose/exposure. General CK/PD characterization alone should not be treated as dose-related unless linked to one of these applications. |
| Model-credibility and reporting appraisal | The appraisal is adapted for mechanistic pharmacological models and should not be presented as a conventional validated risk-of-bias assessment. |
| Not reported | Use only when the information cannot be located in the full text, tables, figures, supplementary files, or referenced model documentation available to the reviewers. |

**Abbreviations:** ABM, agent-based model; B-ALL, B-cell acute lymphoblastic leukemia; CAR-T, chimeric antigen receptor T-cell; CK/PD, cellular kinetic/pharmacodynamic; CRS, cytokine release syndrome; M, mechanistic; ICANS, immune effector cell-associated neurotoxicity syndrome; ICH M15, International Council for Harmonisation M15 guideline on model-informed drug development; LNP, lipid nanoparticle; mPBPK, minimal physiologically based pharmacokinetic; NLME, nonlinear mixed effects; ODE, ordinary differential equation; PBPK/PD, physiologically based pharmacokinetic/pharmacodynamic; PK/PD, pharmacokinetic/pharmacodynamic; QSP, quantitative systems pharmacology; SM, semi-mechanistic.
