# Supplementary material for: Application of Mechanistic Modeling to Support Dose‐Relevant Decision Making for CAR‐T Cell Therapy in Oncology: A Systematic Scoping Review
Source: Clin Transl Sci. 2026 Nov 6;19(11):e70756. doi: 10.1111/cts.70756 (PMC13741396; doi:10.1111/cts.70756)
Supplement: Supplementary file 4 [file CTS-19-e70756-s004.docx]

## Supplementary File S1: Scoping Review Protocol

*Application of mechanistic model-informed dose selection and regimen optimization strategies for CAR-T cell therapy in oncology*

| **Field** | **Protocol details** |
| --- | --- |
| **Protocol version** | Version 1.0 for supplementary material |
| **Protocol date** | 30 Sep 2025 |
| **Review type** | Systematic scoping review |
| **Reporting guidance** | Joanna Briggs Institute methodology and PRISMA-ScR guidance |
| **Protocol registration** | Not formally registered in a systematic review registry (such as PROSPERO); rationale provided below. |
| **Planned review period** | January 2010 to December 2025 |
| **Primary databases** | Ovid MEDLINE, Embase, and Scopus |
| **Supplementary source** | Google Scholar is used as a supplementary source for citation and gray-literature-sensitive checking. |

**1. Background and rationale**

Chimeric antigen receptor T-cell (CAR-T) therapy is a living therapeutic modality characterized by nonlinear cellular kinetic-pharmacodynamic (CK/PD) behavior. Unlike conventional small molecules or biologics, the administered cell dose does not necessarily predict in vivo expansion, persistence, tumor killing, cytokine-mediated toxicity, or clinical response. CAR-T exposure is shaped by tumor burden, antigen engagement, product composition, lymphodepletion, host immune recovery, trafficking, biodistribution, tissue barriers, and immune-mediated toxicity mechanisms.

Mechanistic and semi-mechanistic models can provide biologically interpretable frameworks for linking administered dose, functional cellular exposure, tumor response, toxicity, and regimen components. However, the extent to which such models have been applied to CAR-T dose selection and regimen optimization in oncology has not been consistently mapped. This protocol specifies the methods used to identify, classify, and synthesize original mechanistic and semi-mechanistic modeling studies relevant to this question.

**2. Review question and objectives**

**2.1 Review question**

What mechanistic or semi-mechanistic (i.e.CK/PD, PK/PD, QSP, physiologically based PK/PD, stochastic, or agent-based) models have been developed for CAR‑T cell therapy in oncology, and how have they been applied to dose selection, or regimen optimization?

**2.2 Objectives**

Identify original mechanistic and semi-mechanistic modeling studies applied to CAR-T therapy in cancer indications.

Classify included models by modeling framework, biological resolution, CAR-T platform, indication, target/product context, and development phase.

Map how models inform dose selection, exposure-response interpretation, or regimen optimization strategies.

Summarize biological processes represented across models, including CAR-T expansion, contraction, persistence, trafficking, tumor killing, antigen escape, lymphodepletion, cytokines, CRS, ICANS, exhaustion, and manufacturing/product attributes.

Describe model evaluation, calibration, validation, uncertainty assessment, and credibility considerations using principles aligned with ICH M15, without treating ICH M15 as defining formal M/SM categories.

Identify gaps and recommendations for future regulatory-grade mechanistic CAR-T modeling.

**3. Eligibility criteria**

**3.1 Population-concept-context framework**

| **PCC element** | **Definition for this scoping review** | **Examples** |
| --- | --- | --- |
| **Population** | Cancer patients, cancer-relevant preclinical systems, or virtual populations receiving or representing CAR-T therapy. | B-ALL, non-Hodgkin lymphoma, multiple myeloma, solid tumors, preclinical xenograft or syngeneic tumor models. |
| **Intervention** | Oncology applications of ex vivo or in vivo CAR-T platforms. | Autologous, allogeneic, local/systemic delivery, in vivo mRNA/vector-mediated CAR-T generation. |
| **Concept** | Mechanistic or semi-mechanistic models relevant to CAR-T dose selection, exposure-response interpretation, or regimen optimization. | CK/PD, PK/PD, QSP, PBPK/PD, ODE, stochastic, agent-based, multiscale, or in vivo CAR-T delivery models. |
| **Context** | Dose/regimen/exposure-response | dose selection, dose optimization, dosing, exposure–response, etc. |

**3.2 Inclusion criteria**

Original peer-reviewed primary research published from January 2010 to December 2025.

Study reports development, calibration, simulation, or application of a mechanistic or semi-mechanistic model.

Model is applied to CAR-T cell therapy in an oncology indication or cancer-relevant translational system.

Model of single-target CAR-T cell therapy used for cancer treatment, including autologous or allogeneic ex vivo CAR-T products.

Model includes a biological or mechanistic structure, such as CAR-T expansion/contraction, tumor-cell dynamics, antigen engagement, cytokines, lymphodepletion, trafficking, biodistribution, phenotype transitions, or tumor microenvironment effects.

Model is relevant to at least one of the following: dose selection, exposure-response interpretation, dose-response analysis, regimen optimization, conditioning/lymphodepletion optimization, route/delivery optimization, repeat/fractionated dosing, vector/mRNA dose selection, product composition, or toxicity-monitoring strategy.

Full text available in English.

**3.3 Exclusion criteria**

Review articles, editorials, perspectives, commentaries, conference abstracts without sufficient methodological detail, or regulatory assessments without original model development.

Purely statistical, empirical, or machine-learning models without an explicit mechanistic or biological model structure.

Models focused exclusively on non-cancer indications.

Models of non-CAR-T therapies non-CAR-T cell therapies; dual-targeted, tandem, bicistronic, pooled, cocktail, or other multi-antigen investigational CAR-T products.

Studies describing CK/PD model development or sensitivity analysis only, without dose-related, exposure-response, or regimen-optimization application.

Studies evaluating dual-targeted, tandem, bicistronic, pooled, cocktail, or other multi-antigen CAR-T products were excluded unless model outputs or clinical data were reported separately for a single-target CAR-T product relevant to the review question.

Such studies were categorized as not dose-related at full-text review.

Insufficient reporting to determine model structure, context, or relevance to the review question.

**4. Information sources and search strategy**

**4.1 Information sources**

The planned bibliographic databases were Ovid MEDLINE, Embase, and Scopus. Google Scholar was used as a supplementary source to identify additional records and to check whether recent or cross-disciplinary modeling studies were missed by database searches. Reference lists of included studies and relevant reviews were checked for additional eligible records.

**4.2 Search concepts**

Search terms were developed around three concept domains: CAR-T therapy, mechanistic modeling, and dose/regimen or exposure-response concepts. Controlled-vocabulary terms were combined with free-text terms and adapted for each database.

| **Concept domain** | **Example terms** |
| --- | --- |
| **CAR-T therapy** | chimeric antigen receptor, CAR-T, CAR T, CAR-T cell, adoptive cell therapy, engineered T cell, Tisagenlecleucel, axicabtagene, lisocabtagene, idecabtagene, ciltacabtagene, UCART19 |
| **Mechanistic modeling** | mechanistic model, semi-mechanistic model, cellular kinetics, CK/PD, PK/PD, pharmacodynamic model, pharmacokinetic model, quantitative systems pharmacology, QSP, physiologically based pharmacokinetic, PBPK, ordinary differential equation, ODE, stochastic model, agent-based model, mathematical model, multiscale model |
| **Dose/regimen/exposure-response** | dose selection, dose optimization, dose optimization, regimen optimization, regimen optimisation, exposure response, dose response, cellular exposure, Cmax, AUC, expansion, persistence, lymphodepletion, conditioning, fractionated dosing, repeat dosing, route of administration, delivery, biodistribution, trafficking, vector dose, mRNA dose |
| **Cancer filter** | cancer, oncology, malignancy, leukemia, leukemia, lymphoma, myeloma, solid tumour, solid tumor, glioblastoma, pancreatic cancer, mesothelioma |

**4.3 Draft MEDLINE search template**

The following template was adapted to each database syntax. Final database-specific search strings, search dates, filters, and record counts should be retained in the review audit trail.

((chimeric antigen receptor* OR CAR-T OR CAR T OR CAR-T cell* OR engineered T cell* OR adoptive cell therap*) AND (mechanistic model* OR semi-mechanistic OR cellular kinetic* OR CK/PD OR PK/PD OR pharmacodynamic model* OR quantitative systems pharmacology OR QSP OR PBPK OR physiologically based pharmacokinetic* OR ODE OR ordinary differential equation* OR stochastic model* OR agent-based model* OR mathematical model* OR multiscale model*) AND (dose selection OR dose optimization OR dose optimisation OR regimen optimization OR regimen optimisation OR exposure-response OR dose-response OR expansion OR persistence OR lymphodepletion OR conditioning OR delivery OR trafficking OR biodistribution OR repeat dosing OR fractionated dosing OR vector dose OR mRNA dose) AND (cancer OR oncology OR malignan* OR leukemia OR leukaemia OR lymphoma OR myeloma OR solid tumor OR solid tumour))

**5. Study selection process**

All search results were exported into a reference-management or screening platform, and duplicates were removed using DOI, title, author, journal, and year matching. Screening was conducted in two stages: title/abstract screening followed by full-text review. Two reviewers independently assessed records against the eligibility criteria; disagreements were resolved by consensus, with a third reviewer available if needed.

Full-text exclusion categories were pre‑specified as: review/narrative article, non-mechanistic or empirical model only, not dose-related, non-cancer indication, other cell therapy or non-CAR-T focus, insufficient methodological reporting, duplicate or overlapping report, and not peer-reviewed full research article.

**6. Data charting and extraction**

A standardized data-extraction form was used. Data extraction was performed by one reviewer and verified by a second reviewer. Any uncertainties were resolved by discussion and documented in the extraction log.

Bibliographic details: first author, year, title, journal, DOI.

CAR-T platform: autologous, allogeneic, in vivo CAR-T, local/systemic delivery, product-specific or non-product-specific context.

Development context: preclinical, translational, clinical retrospective, clinical prospective, theoretical/in silico.

Indication and target antigen/product context.

Model class: mechanistic or semi-mechanistic, using pre‑specified structural criteria.

Mathematical framework: ODE, NLME/population CK, QSP, PBPK/PD, stochastic, agent-based, multiscale, translational PK/PD, or hybrid.

Biological processes represented: CAR-T expansion, contraction, persistence, memory/effector/exhausted phenotypes, trafficking, biodistribution, tumor killing, antigen escape, cytokines, CRS/ICANS, lymphodepletion, host immune reconstitution, macrophage/TME components, manufacturing or product attributes.

Dose-selection or exposure-response relevance.

Regimen-optimization relevance: lymphodepletion, scheduling, route, repeat/fractionated dosing, local delivery, vector/mRNA dose, product composition, combination strategy, toxicity intervention.

Model evaluation approach: calibration, goodness-of-fit, visual predictive checks, sensitivity analysis, uncertainty analysis, cross-validation, internal validation, external validation, or simulation only.

Key limitations and applicability domain.

**7. Model classification framework**

Models were classified as mechanistic (M) or semi-mechanistic (SM) for synthesis. This classification was not intended to represent formal regulatory categories and was not defined by ICH M15. Instead, it was used as a pragmatic structural descriptor of the dominant modeling objective and biological resolution.

| **Classification** | **Operational definition** | **Examples of qualifying features** |
| --- | --- | --- |
| **Mechanistic (M)** | Biological mechanisms, system compartments, spatial architecture, or physiologic processes form the dominant organizing structure of the model. | Explicit tumor-immune ODE systems, PBPK/PD trafficking models, agent-based spatial models, stochastic tumor-CAR-T models, cytokine/tumour/immune-interaction systems. |
| **Semi-mechanistic (SM)** | A mechanistic core is combined with empirical, population, statistical, translational, or simplified components that substantially influence inference or prediction. | Population CK models with mechanistic phases, multiscale models calibrated to summary clinical data, QSP/ML hybrids, translational PK/PD models with simplified tumor or toxicity linkage. |

Borderline cases were resolved by reviewer consensus. Classification reflected the dominant model application, not the intrinsic value or regulatory acceptability of the model.

**8. Model-credibility and reporting appraisal**

Because conventional risk-of-bias tools were not designed for mechanistic pharmacological models, the present review used a structured model-credibility and reporting appraisal rather than a conventional risk-of-bias assessment. The appraisal was informed by PROBAST domains, ICH M15 principles, and established CK/PD, QSP, and PBPK model-evaluation concepts.

| **Appraisal domain** | **Adapted focus for this scoping review** | **Example considerations** |
| --- | --- | --- |
| **Data/source relevance** | Appropriateness of data source for intended context of use. | Clinical vs preclinical vs simulation-only; sample relevance; product/indication match. |
| **Inputs and assumptions** | Biological plausibility, transparency, and identifiability of model inputs and parameters. | Tumor burden, antigen expression, lymphodepletion, trafficking, cytokine assumptions, fixed vs estimated parameters. |
| **Endpoints and outputs** | Clinical or translational relevance of model outputs. | Cmax, AUC, expansion, persistence, tumor response, CRS, survival, tumor-site exposure. |
| **Analysis and evaluation** | Adequacy of calibration, verification, validation, sensitivity analysis, and uncertainty assessment. | Goodness-of-fit, VPC, cross-validation, external validation, sensitivity analysis, uncertainty propagation, code/model transparency. |
| **Applicability** | Clarity of the model applicability domain and limits of extrapolation. | Cross-product extrapolation, solid-tumor translation, in vivo CAR-T extrapolation, clinical decision influence. |

**9. Synthesis plan**

Results were synthesized narratively and in tables. No meta-analysis was planned because of expected heterogeneity in model structures, indications, platforms, endpoints, and levels of empirical calibration. Synthesis focused on model purpose, biological process coverage, dose-selection relevance, regimen-optimization relevance, and model evaluation.

Descriptive counts were planned for model class, framework, indication context, platform, evidence level, biological process coverage, dose-selection relevance, regimen-optimization relevance, and evaluation approach.

A taxonomy of model applications was planned to distinguish exposure-response interpretation, tumor burden-informed dosing, antigen/affinity-informed functional dose, lymphodepletion optimization, delivery/route optimization, repeat/fractionated dosing, in vivo vector/mRNA dosing, and toxicity-monitoring models.

Conceptual figures were planned to summarize the model landscape, biological process coverage, and study interrelationships. These figures were intended as conceptual syntheses, not formal bibliometric or network meta-analyses.

Findings were interpreted according to model credibility, context of use, and strength of validation evidence. Models were not assumed to support prospective dose selection unless such use was explicitly evaluated.

**10. Management of amendments and boundary-case decisions**

Search verification and full-text assessment may identify boundary cases. Any such studies were resolved using the pre‑specified eligibility rule requiring explicit relevance to dose selection, exposure-response interpretation, or regimen optimization. Studies that developed CAR-T CK/PD models but did not evaluate dose levels, dose ranges, dose-response, exposure-response, dosing schedules, or regimen strategies were excluded as not dose-related, even if the authors proposed future dose-prediction applications.

Protocol clarifications made during manuscript revision were documented transparently. For example, model-credibility appraisal terminology was revised to avoid presenting an adapted PROBAST-informed approach as a conventional risk-of-bias assessment, and full-text boundary decisions were documented when a study was excluded because its application was CK/PD characterization and sensitivity analysis rather than explicit dose or regimen optimization.

**11. Reporting**

The final review was reported according to PRISMA-ScR. The manuscript includes a PRISMA flow diagram, tables summarizing included study characteristics and model evaluation, a biological process coverage table, and supplementary materials including the PRISMA-ScR checklist and this protocol. Deviations from the protocol and limitations of the review process were described in the manuscript discussion.

**12. Ethics, dissemination, funding, and conflicts of interest**

Ethics approval was not required because the review used published literature and did not involve individual patient-level data collection. Findings were intended for submission to Clinical and Translational Science and dissemination through peer-reviewed publication. Funding sources, conflicts of interest, and author contributions should be reported in the main manuscript. If no specific funding was received, the manuscript should state: “No specific funding was received for this work.”

**Appendix A. Data charting form**

| **Domain** | **Data item** |
| --- | --- |
| **Bibliographic information** | First author; year; title; journal; DOI; country/setting if reported. |
| **Study context** | Cancer indication; target antigen; product or platform; autologous, allogeneic, or in vivo CAR-T; development phase. |
| **Model structure** | Model class; mathematical framework; compartments/states; parameters; model inputs; assumptions. |
| **Dose relevance** | Dose levels; dose-response or exposure-response analysis; dose-selection strategy; functional dose concept; administered vs expanded cell exposure. |
| **Regimen relevance** | Lymphodepletion; route; scheduling; repeat/fractionated dosing; vector/mRNA dosing; delivery/trafficking; combination strategies; toxicity intervention. |
| **Biological process coverage** | CAR-T kinetics; tumor dynamics; antigen escape; cytokines; CRS/ICANS; trafficking; exhaustion; TME; manufacturing/product attributes. |
| **Model evaluation** | Calibration; diagnostics; validation; sensitivity analysis; uncertainty analysis; code availability; applicability domain. |
| **Interpretation** | Clinical/translational relevance; limitations; hypothesis-generating vs decision-supporting use; comments for manuscript synthesis. |

**Appendix B. Planned full-text exclusion categories**

| **Exclusion category** | **Operational definition** |
| --- | --- |
| **Review/narrative article** | No original model development or application. |
| **Non-mechanistic model** | Purely empirical or statistical analysis without biological model structure. |
| **Not dose-related** | No explicit dose-selection, exposure-response, dose-response, regimen, schedule, delivery, or optimization application. |
| **Non-cancer indication** | CAR-T model applied only to non-oncology disease. |
| **Other cell therapy / not CAR-T** | Model not specific to CAR-T therapy. |
| **Insufficient reporting** | Full-text information inadequate to classify the model or assess relevance. |
| **Duplicate/overlap** | Duplicate publication or substantially overlapping report without additional eligible model application. |

**References**

International Council for Harmonisation. ICH M15: General Principles for Model-Informed Drug Development. Final guideline.

Peters MDJ, Godfrey C, McInerney P, Munn Z, Tricco AC, Khalil H. Chapter 11: Scoping Reviews. In: JBI Manual for Evidence Synthesis. 2020.

Tricco AC, Lillie E, Zarin W, et al. PRISMA Extension for Scoping Reviews (PRISMA-ScR): Checklist and explanation. Ann Intern Med. 2018;169:467-473.

Page MJ, McKenzie JE, Bossuyt PM, et al. The PRISMA 2020 statement: an updated guideline for reporting systematic reviews. BMJ. 2021;372:n71.

Wolff RF, Moons KGM, Riley RD, et al. PROBAST: A tool to assess the risk of bias and applicability of prediction model studies. Ann Intern Med. 2019;170:51-58.
